# Breaking Bad News: Dynamic Molecular Mechanisms of Wound Response in Plants

**DOI:** 10.3389/fpls.2020.610445

**Published:** 2020-12-08

**Authors:** Isaac Vega-Muñoz, Dalia Duran-Flores, Álvaro Daniel Fernández-Fernández, Jefri Heyman, Andrés Ritter, Simon Stael

**Affiliations:** ^1^Laboratorio de Ecología de Plantas, CINVESTAV-Irapuato, Departamento de Ingeniería Genética, Irapuato, Mexico; ^2^Department of Plant Biotechnology and Bioinformatics, Ghent University, Ghent, Belgium; ^3^VIB-UGent Center for Plant Systems Biology, Ghent, Belgium; ^4^Department of Biomolecular Medicine, Ghent University, Ghent, Belgium; ^5^VIB-UGent Center for Medical Biotechnology, Ghent, Belgium

**Keywords:** wound response, damage, damage-associated molecular pattern, systemic signaling, herbivory, jasmonic acid, regeneration

## Abstract

Recognition and repair of damaged tissue are an integral part of life. The failure of cells and tissues to appropriately respond to damage can lead to severe dysfunction and disease. Therefore, it is essential that we understand the molecular pathways of wound recognition and response. In this review, we aim to provide a broad overview of the molecular mechanisms underlying the fate of damaged cells and damage recognition in plants. Damaged cells release the so-called damage associated molecular patterns to warn the surrounding tissue. Local signaling through calcium (Ca^2+^), reactive oxygen species (ROS), and hormones, such as jasmonic acid, activates defense gene expression and local reinforcement of cell walls to seal off the wound and prevent evaporation and pathogen colonization. Depending on the severity of damage, Ca^2+^, ROS, and electrical signals can also spread throughout the plant to elicit a systemic defense response. Special emphasis is placed on the spatiotemporal dimension in order to obtain a mechanistic understanding of wound signaling in plants.

## Introduction

Plants are especially susceptible to damage as they are unable to run away when facing danger. Wounds can originate from harsh weather conditions (e.g., strong wind, hail, fire, and frost), physical damage (e.g., trampling), exposure to chemicals (e.g., DNA damage and toxic substances), or biotic attack (e.g., microbes and herbivores). Damage can range in severity from single cell death to complete removal of organs and in duration from single events to repeated injury, for example, from chewing insects. In the lab, mechanical damage can be rather “clean” as in cutting with a sharp razor blade, application of pin pricks, and laser-mediated wounding, or “messy” by bruising tissue with pinches of a forceps or hemostat. We define here “wound” (wounding, wound-induced, etc.) as a general term, while the type of damage that produced the wound can be further specified, such as mechanical- or herbivore-induced damage.

In contrast to metazoans, plants do not rely on a dedicated nerve system or mobile immune cells to sense or respond to wounds. Nevertheless, plants have evolved efficient mechanisms to perceive wounds and mount an appropriate defense response. Each plant cell has the ability to transduce a signal to its neighboring cells *via* damage-associated molecular patterns (DAMPs; previously reviewed in Heil and Land, 2014). Depending on the severity of the damage in size or location (e.g., leaf midvein; Kiep et al., 2015; Toyota et al., 2018), the complete plant can be alerted through a systemic signal, spreading from local to distal tissues that comprises waves of hydraulic, electrical, calcium (Ca^2+^), and reactive oxygen species (ROS) signals, and the perception of wound-related hormones, such as jasmonic acid (JA), ethylene, or abscisic acid (ABA). Once activated, chemical defenses, such as the production of phytoalexins and other secondary metabolites, or structural defenses, such as increased production of trichomes and strengthening of cell walls, can protect the plant from reoccurring damage (Agrawal, 1998; Maffei et al., 2007b). Several aspects of the wound response are conserved with metazoans, including the release of certain DAMPs, Ca^2+^, and ROS signaling. Other traits are plant-specific, such as the production of wound hormones and release of wound-induced volatiles. Some responses share similarities, such as the production of oxylipins (JA in plants and prostaglandins or leukotrienes in metazoans) and activation of membrane localized receptors by DAMPs and downstream phosphorylation cascades to activate defense gene expression (previously reviewed in León et al., 2001; Maffei et al., 2007a; Heil and Land, 2014; Savatin et al., 2014).

The ability to sense and appropriately respond to wounds is crucial for survival. On the one hand, a defective or overwhelmed defense response leads to increased plant mortality (Agrawal, 1998), especially what concerns the replenishment of stem cells and regeneration of organs in the root and shoot apical meristems and cambium (Sena et al., 2009; Heyman et al., 2013; Efroni et al., 2016). On the other hand, mechanisms are in place to prevent plants from overreacting to wounds and, when compromised, can lead to uncontrolled spread of cell death (Cui et al., 2013) or hypersensitivity to wounding (Zhang et al., 2019). Wound healing and defense responses can prevent excessive water loss (Consales et al., 2012; Cui et al., 2013; Becerra-Moreno et al., 2015), attenuate pathogen infection (Tarr, 1972; Lulai and Corsini, 1998; Zhou et al., 2020), and deter herbivores (previously reviewed in Erb and Reymond, 2019).

In nature, wounds are likely pervasive even when not visible to the naked eye and provide easy access sites for some pathogens, especially wound parasites such as wood rot and canker fungi (previously reviewed in Tarr, 1972). Pathogen colonization is prevented by wound healing processes, such as production of cork, callus, resin, or gum, and relies on rapid sealing of wounds (Lulai and Corsini, 1998). Furthermore, the immune system is activated in response to wounding (Savatin et al., 2014; Zhou et al., 2020). Therefore, wound-induced resistance can inhibit pathogen growth, for example, in the local resistance to *Botrytis cinerea* (Chassot et al., 2008; García et al., 2015), although it likely depends on environmental circumstances, such as high humidity (L’Haridon et al., 2011) and the natural genetic variation of the host plant (Coolen et al., 2019). Furthermore, effective colonization of the wound depends on the timing of contact with the pathogen (present before wounding or only after) and degree of wounding (Lulai and Corsini, 1998; Chassot et al., 2008). Therefore, pathogen entry *via* wounds merits further investigation and should be evaluated in a case-by-case scenario. Both microbes and invertebrate herbivores will attempt to subvert wound-induced defense responses. Interaction with chewing or sucking insects is further complicated as both insects and insect-borne microbes produce elicitors and suppressors of plant defense, in which JA signaling is often the target (previously reviewed in Basu et al., 2018). Due to the co-evolution of plants and pests, it is to be expected that every wound response is a potential target for suppression by pathogens and herbivores. Therefore, interactions of wounds with biotic challenges pose interesting cases, where wound responses can be enhanced or subverted, and some examples will be highlighted throughout this review.

Studies of wound response in plants present a long tradition of research. Whereas the first studies were mainly descriptive (Bloch, 1941; Lipetz, 1970), in the last decades, molecular mechanisms are increasingly becoming clear (León et al., 2001; Maffei et al., 2007a; Savatin et al., 2014). For information on wound healing and mitigation of damage in post-harvest processes in vegetables and fruit, we refer to specific literature (Cisneros-Zevallos et al., 2014; Lulai et al., 2016; Saltveit, 2016; Iakimova and Woltering, 2018; Hussein et al., 2020). This review provides a broad overview of the recent developments in molecular mechanisms with a focus on spatiotemporal dynamics in order to gain mechanistic understanding and to address open questions in the field of wound response in plants.

## Local Vs. Systemic Wound Signaling

Wound signaling can be divided in a local and systemic response. Cells at the site of injury can be completely destroyed or bruised (Iakimova and Woltering, 2018) and, at least in leaves, cell death ensues at the timescale of hours to days in 2–3 cell layers away from the site of injury (Cui et al., 2013; Iakimova and Woltering, 2018). Together with the local deposition of lignin, callose, and phenolics, cell death likely functions as a physical barrier to seal-off the injury and protects the adjacent intact tissue (Savatin et al., 2014; Iakimova and Woltering, 2018). DAMPs released from wounds signal the surrounding intact cells *via* Ca^2+^, ROS, phosphorylation, and electrical signaling to mount defense gene expression. Most likely, direct physical responses, such as changes in mechanical forces and cell pressure surrounding the wound, play a pronounced signaling role, although these are largely unknown (Routier-Kierzkowska et al., 2012; Hoermayer et al., 2020). In parallel and depending on the severity of damage, systemic signals are propagated from the wound site to the rest of the plant, comprising leaf-to-leaf, root-to-root, leaf-to-root, and root-to-leaf signaling. Local and systemic responses are inherently linked at least through Ca^2+^, ROS, and electrical signaling, and, where information is available, links will be highlighted throughout the review.

### The Ins and Outs of DAMPs Generation and Recognition

Plants have evolved mechanisms that allow them to respond quickly to wounding and to distinguish the self from the non-self (Heil and Land, 2014; Savatin et al., 2014). Plant innate immunity relies on cell surface receptors that allows activation of defense responses *via* the recognition of conserved exogenous pathogen-derived (non-self) or endogenous (self) danger signals by transmembrane pattern-recognition receptors (PRRs). These conserved danger signals are also termed as pathogen-associated molecular patterns [PAMPs; also named microbe-associated molecular patterns (MAMPS) in the literature] for the non-self-signals and DAMPs for the self-signals (Choi and Klessig, 2016). In this review, we will discuss recent progress on several prominent DAMPs and their links to wound response, while for an extensive overview of DAMPs, we refer to recent excellent reviews (Choi and Klessig, 2016; Duran-Flores and Heil, 2016; Gust et al., 2017; Hou et al., 2019).

#### Primary/Constitutive and Secondary/Inducible DAMPs

**Figure 1:**
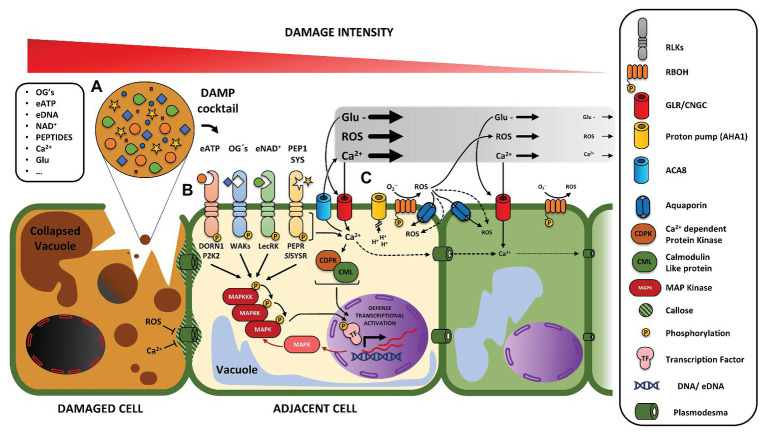
Overview of local damage at early time-points following signaling processes. **(A)** Damaged cells suffer fragmentation of their cellular components, releasing a mixture of different damage-associated molecular patterms [DAMPs (DAMP cocktail)] to the surrounding environment. Reactive oxygen species (ROS) and Ca^2+^ contribute to plasmodesmata blockage and later accumulation of callose. **(B)** DAMPs are perceived by specific receptors, generally receptors like kinases in the plasma membrane. DAMP sensing is normally accompanied by hallmark signal transduction events, such as MAPK phosphorylation cascades, that result in transcription factor phosphorylation and modulation of defense gene expression. **(C)** Parallel to local perception by receptors, certain signals such as Ca^2+^, ROS, and glutamate can travel from the wound site in a distance-dependent gradient along the apoplast. GLR and CNGC channels can be activated by DAMPS such as glutamate and Pep1. Intracellular Ca^2+^ serves as a component to activate calcium-dependent protein kinases (CDPK) and calmodulin-like proteins (CML), which contribute to transcriptional responses. Together, oxygen radicals are generated locally in the extracellular space and transformed to more stable ROS species by RBOHs, thus adjusting the ROS wave signal.

Wounding either by mechanical damage, herbivores, or microbial infections results in disruption of plant tissue and subsequent release of intracellular molecules and cell wall-associated molecules into the apoplastic space (Mithöfer and Boland, 2012; Choi and Klessig, 2016; Duran-Flores and Heil, 2016; Figure 1A). Herbivores destroy plant tissues during feeding and/or by chemical modification while microbial infection-induced plant damage is often caused by deleterious activities of microbial hydrolytic enzymes or toxins (D’Ovidio et al., 2004; Horbach et al., 2011). Molecules released passively upon host damage conform to the definition of “classical” or primary DAMPs (Matzinger, 1994), which are molecules that have a physiological role during homeostasis but indicate damage when they appear outside the cell. Examples are ATP, cell wall fragments occasioned by wounding or pathogen derived cell wall degrading enzymes, or fragmented DNA caused by pathogen DNases (Claverie et al., 2018; Hadwiger and Tanaka, 2018; Huang et al., 2019; Jewell and Tanaka, 2019). Location is important, as these DAMPs are invisible to the immune system during homeostasis and are passively exposed to the extracellular environment, thereby acting as early and general activators of the plant immune system (Vénéreau et al., 2015; Choi and Klessig, 2016). Thus, primary DAMPs are not linked to biosynthesis or secretion from undamaged cells. The secondary or inducible DAMPs are endogenous molecules actively produced or modified during cell death and function exclusively as signals. They can be secreted passively or actively upon wounding or microbial infection by either damaged or undamaged cells and include, for example, small signaling peptides (Gust et al., 2017; Li et al., 2020). Details about the temporal activation of the signaling molecules and hormones upon perception of DAMPs mentioned in the text can be retrieved in Table 1.

**Table 1:** Timing and localization of DAMP release/generation and wound responses in plants.

DAMP	Receptor	Release/generation	Localization	Response	Source	Response time	Plant species	References
Ogs	WAK1/2	< 4 h (Polygalacturonase; Bergey et al., 1999)	-	Ca^2+^	Ex	2 min	Tobacco	Chandra and Low, 1997
				ROS	Ex	2 h	Tobacco	Bellincampi et al., 2000
				ROS	Ex	15 min	*Arabidopsis*	Galletti et al., 2008
				MAPK	Ex	3 min	*Arabidopsis*	Denoux et al., 2008
				NO	Ex/*in vivo*	30 min	*Arabidopsis*	Rasul et al., 2012
				Callose	Ex	18 h	*Arabidopsis*	Denoux et al., 2008
eATP	P2K1	< 1 min (Song et al., 2006)	Extracellular	Ca^2+^	Ex	30–40 s	*Arabidopsis*	Tanaka et al., 2010
				Ca^2+^	Ex/*in vivo*	1–2 min	*Arabidopsis*	Demidchik et al., 2009
				ROS	Ex/*in vivo*	15 s	*Arabidopsis*	Demidchik et al., 2009
				ROS	Ex/*in vivo*	5 min	Medicago	Kim et al., 2006
				JA	Ex/*in vivo*	24 h	Tomato	Wu et al., 2012
				Et	Ex/*in vivo*	24 h	Tomato	Wu et al., 2012
NAD(P)+	LecRK-1.8/VI.2	< 20 min (Zhang and Mou, 2009)	Extracellular	SA	Ex	4 h	*Arabidopsis*	Wang et al., 2017
				PR genes	Ex/*in vivo*	24 h	*Arabidopsis*	Zhang and Mou, 2009
				SA	Ex	-	*Arabidopsis*	Zhang and Mou, 2009
HMGB3	-	24 h (Choi et al., 2016)	Apoplast	MAPK	Ex/*in vivo*	15 min	*Arabidopsis*	Choi et al., 2016
				Callose	Ex/*in vivo*	15 h	*Arabidopsis*	Choi et al., 2016
DNA	-	-	-	Ca^2+^	Ex	30 min	Maize	Barbero et al., 2016
				Ca^2+^	Ex	30 min	Lima Bean	Barbero et al., 2016
				ROS	Ex	2 h	Common Bean	Duran-Flores and Heil, 2018
				MAPK	Ex	30 min	Common Bean	Duran-Flores and Heil, 2018
Systemin	SYR1/2	3–4 h (mRNA; McGurl et al., 1992)	Intracellular	Ca^2+^	Ex	2 min	Tomato	Moyen et al., 1998
				ROS	Ex/*in vivo*	4 h	Tomato	Orozco-Cardenas and Ryan, 1999
				MAPK	Ex/*in vivo*	2 min	Tomato	Stratmann and Ryan, 1997
		18 h (prosystemin; Narváez-Vásquez and Ryan, 2004)	Phloem	Et	Ex/*in vivo*	30 min	Tomato	O’Donnell et al., 1996
				JA	Ex/*in vivo*	15 min	Tomato	Narváez-Vásquez et al., 1999
				PI	*in vivo*	1 h	Tomato	Howe et al., 2000
Inceptin	INR	-	-	JA	Ex/*in vivo*	30 min	Cowpea	Schmelz et al., 2007
				Et	Ex/*in vivo*	120 min	Cowpea	Schmelz et al., 2007
				SA	*Ex*/*in vivo*	240 min	Cowpea	Schmelz et al., 2007
Glutamate	GLR	< 1 min (Toyota et al., 2018)	Vasculature	Ca^2+^	*in vivo*	1 min	*Arabidopsis*	Toyota et al., 2018
				Ca^2+^	Ex	1 min	*Arabidopsis*	Shao et al., 2020
				SA	Ex	6 h	*Arabidopsis*	Goto et al., 2020
				JA	Ex	7 h	*Arabidopsis*	Goto et al., 2020
AtPep1	AtPEPR1/2	0.5–5 min (Hander et al., 2019)	Intracellular	Ca^2+^	Ex	40 s	*Arabidopsis*	Ranf et al., 2011
				MAPK	Ex	2 min	*Arabidopsis*	Ranf et al., 2011
				ROS	Ex	4 min	*Arabidopsis*	Flury et al., 2013
AtPep3	AtPEPR1	24 h (Yamada et al., 2016; Engelsdorf et al., 2018)	Extracellular	ROS	Ex	2 h	Rice cells	Shinya et al., 2018
				MAPK	Ex	15 min	Rice cells	Shinya et al., 2018
				JA	Ex	3 h	Rice cells	Shinya et al., 2018
				JA	*in vivo*	4 h	*Arabidopsis*	Klauser et al., 2015

Ex, exogenous application; ROS, reactive oxygen species; MAPK, mitogen-activated protein kinase; NO, nitric oxide; JA, jasmonic acid; SA, salicylic acid; Et, ethylene, PR, pathogenesis related; and PI, protease inhibitors.

#### Oligogalacturonides

Cell wall integrity is crucial for plant growth and development as well as in preventing wounding and pathogen attack (Bellincampi et al., 2014). Perception of an altered cell wall integrity is proposed to be a key event during wounding (Nühse, 2012; Wolf et al., 2012; Wolf, 2017), although experimental evidence is lacking so far. Oligogalacturonides (OGs) are released from the plant cell walls from the fragmentation of homogalacturonan, the main component of pectin, either by endogenous wound-induced polygalacturonases or during infection by microbial polygalacturonases (Savatin et al., 2014). OGs are relatively immobile in the plant vascular system and may act as a local signal; however, because polygalacturonase activity is induced systemically in response to wounding, OGs may amplify responses in undamaged leaves (Table 1; Bergey et al., 1999). The size of OG fragments is a major factor dictating their elicitor activity, being OGs with a degree of polymerization between 10 and 15 the most active while shorter oligomers are inactive. OG-induced defense responses include production of ROS (Bellincampi et al., 2000), mitogen-activated protein kinase (MAPK) activation (Denoux et al., 2008), nitric oxide (NO; Rasul et al., 2012), and upregulation of phytoalexins and glucanase (Davis and Hahlbrock, 1987), chitinase (Broekaert and Peumans, 1988), and callose (Denoux et al., 2008; Galletti et al., 2008). In tomato, OGs induce the accumulation of a protease inhibitor, which is effective against insect herbivores (Moloshok et al., 1992; Ryan and Jagendorf, 1995). The *Arabidopsis* wall-associated kinase 1 (WAK1) has been described as an OG receptor. *In vitro* studies have demonstrated that WAK1 binds to polygalacturonic acid, pectins, and specifically to OGs with a degree of polymerization over nine moieties (Decreux and Messiaen, 2005; Cabrera et al., 2008; Brutus et al., 2010). Furthermore, gene expression studies indicate that WAK1 is upregulated by wounding and exogenous application of OGs (Wagner and Kohorn, 2001; Denoux et al., 2008; Ferrari et al., 2013). Alterations in the expression of WAK1 and of its interactors disturb the local response to wounding (Gramegna et al., 2016; De Lorenzo et al., 2018). Hyperaccumulation of OGs may affect growth of the whole plant, eventually leading to cell death (Benedetti et al., 2015), suggesting that OGs play a role in the growth-defense trade-off (Huot et al., 2014). Hence, plants limit the hyperaccumulation of OGs by a battery of at least four *Arabidopsis* enzymes belonging to the family of the so-called berberine-bridge enzyme (BBE-like) proteins (Daniel et al., 2017). BBE-like proteins specifically oxidize OGs and produce oligosaccharides that reduce the ability to induce expression of defense genes, ROS burst, and deposition of callose (Benedetti et al., 2018). Similarly, cellodextrines, degradation products of cellulose, trigger a signaling cascade during immunity, and oxidation by other BBE-like proteins impairs elicitor activity (Locci et al., 2019). Recently, an application of OGs accelerated mechanical wound healing in tomato fruit *via* elicitation of callose deposition, defense gene expression, lignin biosynthesis, and phenylalanine ammonia-lyase activity around the wound in a Ca^2+^ signaling-dependent manner (Lu et al., 2021).

#### Extracellular ATP, NAD^+^, and NADP^+^

Adenosine-5-triphosphate (ATP) represents the universal energy source for metabolic processes. During wounding, ATP is released immediately from the cytoplasm to the outside of the cell (Table 1). This extracellular ATP (eATP) is recognized as a DAMP and has been reported to activate defense responses in fungi, mammals, and plants (Medina-Castellanos et al., 2014; Tripathi and Tanaka, 2018; Roux and Clark, 2019). Concentrations of approximately 40 uM eATP have been measured in the extracellular fluid present at wound sites within 3 min following damage to *Arabidopsis* leaves, which are sufficient to initiate an immune response (Song et al., 2006). In mammals, eATP is recognized by plasma membrane-localized P2-type purinergic receptors. In *Arabidopsis*, eATP, as a DAMP, is sensed by the L-type lectin receptor kinases P2K1 (also known as does not respond to nucleotides 1 or DORN1) and P2K2 at concentrations well below 40 uM (Choi J. et al., 2014; Pham et al., 2020). Transcriptional studies of a *p2k1* mutant in the absence of stimuli revealed only 21 differentially expressed genes compared to the wild type. Such a small number could indicate that P2K1-mediated eATP signaling does not play a major role in growth and development under homeostasis (Jewell and Tanaka, 2019). Approximately 60% of the genes induced by eATP are also induced by wounding, indicating that eATP plays an important role in response to wounding (Choi J. et al., 2014). Furthermore, physical damage in plants that overexpress *P2K1* enhanced upregulation of wound-induced gene expression, while this expression is notably reduced in the *p2k1-3* mutant (Choi J. et al., 2014). Early eATP induced responses include membrane depolarization, Ca^2+^ influx, ROS formation, malondialdehyde production, enzymatic activity (catalase and polyphenol oxidase), JA, and ethylene biosynthesis (Kim et al., 2006; Tanaka et al., 2014; Tripathi et al., 2018; Wang Q.-W. et al., 2019). eATP treatment of wounded tissue resembles a JA-dependent defense response, resulting in the secretion of extrafloral nectar in lima bean to attract predators of herbivores (Heil et al., 2012). Induced immunity by eATP has been reported at the phenotypic level in response to bacteria (Chivasa et al., 2009; Chen et al., 2017), necrotrophic fungi (Tripathi et al., 2018), and herbivores (Heil et al., 2012). ATP receptors, *p2k1-3*, *p2k2* single mutant, and *p2k1p2k2* double mutants, are more susceptible to bacterial infection compared to the wild type, whereas *P2K2* complemented lines showed no difference to the wild type and ectopically expressed *P2K2* showed elevated resistance to bacterial infection (Pham et al., 2020). Saliva from *Helicoverpa zea* larvae degrades eATP from tomato leaves *via* multiple ATPases. The ATPases also suppress wound-induced expression of glandular trichomes in newly forming leaves, thus acting as a herbivore effector suppresses eATP induced wound response (Wu et al., 2012). Similarly, mechanical stress can be coupled to the release of extracellular ATP. In fact, it plays an important role in the root avoidance response, where sensing mechanical stimulation elicited by contacting an object triggers root growth, allowing it to avoid and overcome physical obstacles. Exogenously applied ATP changes the sensitivity of the root tip to the growth-regulating plant hormone auxin and reduces shootward auxin transport (Tanaka et al., 2010). Plants respond to eATP in a dose-dependent manner. Constitutive levels of eATP appear to be essential, as depletion can trigger cell death (Chivasa et al., 2005), while low (30 μM) or moderate (150 μM) doses of eATP can stimulate or suppress cell elongation, respectively (Clark et al., 2010). High eATP doses (>500 μM) reduce cell viability and can trigger programed cell death (Sun et al., 2012; Deng et al., 2015). While there is no direct evidence that eATP alone affects plant growth/regeneration after wounding, data suggest that a combination of several cues like DAMPS, PAMPS, ion/osmolyte concentrations, or mechanical stresses trigger a defense and regeneration response (Marhavý et al., 2019; Shanmukhan et al., 2020; Zhou et al., 2020).

NAD^+^ and NADP^+^, as di-nucleotides and similarly to ATP acting as a classical cofactor, can be released to the environment after wounding, through membrane leakage or active processes such as exocytosis in animal model species (Haag et al., 2007). In *Arabidopsis*, an application of exogenous NAD^+^ (eNAD^+^) and eNADP^+^ is sufficient to induce salicylic acid (SA) accumulation, expression of pathogenesis-related (PR) genes, and resistance to pathogens (Zhang and Mou, 2009; Wang et al., 2017). A lectin receptor kinase, LecRK-I.8, was found to be partially responsible for eNAD^+^ perception (Wang et al., 2017), while LecRK-VI.2 has been proposed as a receptor of both eNAD^+^ and eNADP^+^ (Wang C. et al., 2019). Transcriptome analyses suggest that eNAD+ signaling upregulates genes involved in PAMP triggered immunity and SA pathways but suppresses genes of the JA and ethylene pathways, which are more related to wounding (Wang et al., 2017). However, eNAD^+^ and eNADP^+^ leak into the extracellular space during mechanical wounding and pathogen-induced hypersensitive response in concentrations high enough to induce the latter responses (Table 1), raising the possibility that they act as DAMPs (Zhang and Mou, 2009; Wang C. et al., 2019).

#### High Mobility Group Box Proteins

High mobility group box (HMGB) proteins are highly conserved chromatin-architecture regulators found in all eukaryotes, including plants. Mammalian HMGB1 was one of the first DAMPs to be identified and is extensively characterized and considered a primary DAMP (Choi and Klessig, 2016). Briefly, human HMGB1 binds in the nucleus to DNA, facilitating nucleosome formation and transcription factor binding (Thomas and Travers, 2001; Lotze and Tracey, 2005). Upon its release outside the cell, it can be recognized by various cell surface receptors (Heil and Vega-Muñoz, 2019). In metazoans, HMGB1 facilitates tissue repair and healing by promoting the switch of macrophages to a tissue-healing phenotype (Bianchi et al., 2017). Based on their nuclear location and domain structure, plant HMGB-type proteins might function in a similar way to mammalian HMGB1. The presence of extracellular AtHMGB3 raised the possibility that, similar to the classical role of HMGB1 as mammalian DAMP, it serves in a similar way in plants (Choi et al., 2016). Notably, AtHMGB2/3/4 are present in the cytoplasm as well as in the nucleus. Cytoplasmic functions for these proteins have not yet been reported; however, it is theorized that the cytosolic subpopulation might have easy access to the apoplast after wounding in comparison to the ones found in the nucleus (Pedersen et al., 2010; Choi and Klessig, 2016). To our knowledge, there is no evidence that AtHMGB3 is secreted into the apoplast, so extracellular AtHMGB3 is most likely the result of cell membrane rupture. In fact, tissue damage during *Botrytis cinerea* infection causes the release of AtHMGB3 to the apoplast after 24 h of inoculation, whereas a control protein, histone H3, only appears in the total leaf and nuclear extracts at that timepoint, suggesting that AtHMGB3 is released early during necrosis (Table 1; Choi et al., 2016). Exogenous application of AtHMGB3 induces innate immune responses like MAPK activation, defense gene expression, callose deposition, and enhanced resistance to pathogen infection (Choi et al., 2016).

#### DNA

Plant immunity can be activated upon the sensing of DNA. Cell death during pathogen infection or abiotic stresses leads to DNA fragmentation (Ryerson and Heath, 1996; Kuthanova et al., 2008). Fragmented DNA can be exposed to the apoplast and function as a DAMP. Several recent studies have found evidence that the host-derived fragmented DNA (<700 bp) triggers early plant defense responses, such as membrane depolarization, Ca^2+^ influx, ROS production, and MAPK activation, and eventually induces changes in CpG methylation, and increases plant resistance to pathogen infections (Wen et al., 2009; Barbero et al., 2016; Duran-Flores and Heil, 2018; Vega-Muñoz et al., 2018). Intriguingly, the ability of non-self-derived DNA to trigger an immune response is lower or undetectable than the ones induced by self-derived DNA (Duran-Flores and Heil, 2018), suggesting a species-specific perception mechanism that discriminates self-derived DNA from non-self DNA. To date, no DNA receptor has been identified in plant cells, and none of the receptors that are known from mammals discriminate between self and non-self DNA (Heil and Vega-Muñoz, 2019). Extracellular DNA present on plant root tips is required for defense against a necrotrophic fungus (Wen et al., 2009), and it was recently reported that secreted DNases by a fungal pathogen (*Cochliobolus heterostrophus*) and a herbivore (*Laodelphax striatellus*) serve as effectors that suppress DNA-dependent plant immunity, reinforcing the biological relevance of DNA as a DAMP in plants (Huang et al., 2019). Importantly, to the best of our knowledge, there is no evidence for wound-induced DNA release to the apoplast in plants. However, based on evidence of DNA release in mammalian studies (Marichal et al., 2011; Pottecher et al., 2019; Gong et al., 2020), it is anticipated to be similarly present in plants, but requires further investigation.

Links between the DNA damage response (DDR), cell cycle, programed cell death, and immunity have emerged in recent years (Song et al., 2011; Yan et al., 2013; Hu et al., 2016; Johnson et al., 2018). Depending on the cell type and the severity of the DNA damage, different cellular responses are triggered. In mammals, mild DNA damage leads to cell-cycle arrest, whereas severe and irreparable damage leads to senescence or cell death programs (Surova and Zhivotovsky, 2013). In plants, the presence of damage-inducing agents or defective DNA repair leads to aberrant organogenesis and development, as well as loss of biomass (Hu et al., 2016). In addition, other reports link DDR to the activation of the plant immune system. Pathogen infection triggers the production of SA, which in turn induces DNA damage that can be sensed by DNA repair mechanisms to the site of DNA damage for repair or activation of defense gene expression (Yan et al., 2013). Suppressor of gamma response 1 (SOG1) is a transcription factor of the NAC family and is a central regulator of the plant DDR (Yoshiyama et al., 2009). DDR has been reported to play an essential role for plants to cope with various environmental stresses (Yan et al., 2013; Hong et al., 2017; Ogita et al., 2018). *sog1-1* mutants are deficient in DDR and immune response, while *SOG1* overexpression in the presence of zeocin, a double-strand DNA break agent, enhances DDR, the expression of genes involved in chitin response, and fungal resistance (Yoshiyama et al., 2020). Ethylene response factor 115 (ERF115) is a transcription factor that is upregulated in meristematic cells that are positioned adjacent to dead ones in the root tip. Severe stress conditions may cause irreparable DNA damage resulting in cell death, followed by the induction of regeneration in an ERF115-dependent manner (Heyman et al., 2016, 2018). Besides SA, specific agents that cause DNA alterations (e.g., DNA helical distortion, intercalation, base substitutions, methylation, etc.) enhance defense gene expression. DNA damage and resulting chromatin structural changes may be a central mechanism in initiating defense gene transcription during nonhost resistance (Hadwiger and Tanaka, 2018). Links between DNA damage, immunity, and regeneration have been emerging in the last years, yet, it remains unclear how DNA is sensed as no formal DNA receptors have been reported.

#### Systemin and Other Small Signaling Peptides

Small signaling peptides can be generated as the product of two activities: by transcriptional responses inducing small open reading frames coding for small peptides or by proteolytic processing of precursor proteins (Tavormina et al., 2015; Hou et al., 2019). Proteolytic cleavage generates peptides that are able to alarm surrounding tissues about the imminent stress when perceived *via* plasma membrane associated receptor-like kinases (Wang and Irving, 2011; Stührwohldt and Schaller, 2019). Although experimental evidence has accumulated over the last years, the functions, receptors, mode of actions, and proteases that liberate the peptides from their precursors are still largely unexplored (Tavormina et al., 2015; Schardon et al., 2016; Hander et al., 2019; Chen et al., 2020).

Systemin was the first reported extracellular peptide that induces defense signaling in plants (Pearce et al., 1991). From its precursor, prosystemin, mature systemin (18 amino acids in length) is partially processed by the cysteine protease phytaspase and released into the apoplast during mechanical damage (Beloshistov et al., 2018). Phytaspase might get access to intracellular prosystemin *via* cellular disruption or *via* active delocalization upon programed cell death (Chichkova et al., 2010; Beloshistov et al., 2018). *Prosystemin* expression is low in unwounded leaves and increases several fold, peaking around 4 h after wounding (McGurl et al., 1992). Prosystemin accumulates mainly in the cytosol and nucleus of phloem parenchyma cells (Narváez-Vásquez and Ryan, 2004). Systemin specifically binds its receptors Systemin receptor 1 and 2 (SYR1 and SYR2), which is sufficient to induce the typical response including a ROS burst, ethylene production, and the expression of two wound induced proteinase inhibitors in tomato (Wang et al., 2018). Functionally related peptides are the hydroxyproline-rich glycopeptide systemins. Repetition of these peptides found in the polypeptide precursor proHypSys is thought to magnify the intensity of the wound response once processed (Pearce, 2011). These genes encode different peptides for tobacco, petunia, tomato, and sweet potato but have in common that they are transcriptionally responsive to wounding and/or JA, and above all, they induce similar responses as systemin (Pearce et al., 2001, 2007; Ryan and Pearce, 2003; Ren and Lu, 2006; Chen et al., 2008). Systemin has only been identified in Solanaceae species (Pearce et al., 1991). However, peptides similar to systemin have been identified in other plant species, such as HypSys, Peps, GmSubPep, GmPep914, GmPep690, and PIPs, that act as DAMPs, eliciting high levels of proteinase inhibitors, JA, and release of volatiles within minutes of exogenous peptide application (Albert, 2013; Huffaker et al., 2013; Hou et al., 2019).

Protein elicitor peptide 1 (Pep1) was extracted from *Arabidopsis thaliana* lysates (Huffaker et al., 2006) and is the founding member of a gene family in *Arabidopsis* of eight with various expression patterns under normal and biotic or abiotic stress conditions (Huffaker and Ryan, 2007; Bartels et al., 2013; Bartels and Boller, 2015). Peps are encoded in the C-terminus of their precursors, PROPEPs, which are found in both monocots and dicots (Huffaker et al., 2013; Lori et al., 2015) and play multiple roles in defenses to pathogens, herbivores, and abiotic stresses (Ross et al., 2014; Klauser et al., 2015; Yamada et al., 2016; Engelsdorf et al., 2018; Lee et al., 2018; Nakaminami et al., 2018; Zheng et al., 2018; Jing et al., 2020; Zhang and Gleason, 2020). Ca^2+^ release in mechanically damaged cells activates the cysteine protease metacaspase4 (MC4) to cleave Pep1 from its precursor PROPEP1 within 5 min after wounding (Hander et al., 2019; Zhu et al., 2020). Metacaspases are evolutionary conserved proteases with nine members in the *Arabidopsis* gene family (Klemenčič and Funk, 2018; Minina et al., 2020) of which various metacaspases can cleave different PROPEPs (Hander et al., 2019; Shen et al., 2019). Cleavage of PROPEP1 seems to be essential for release of Pep1 from the tonoplast (Bartels et al., 2013; Hander et al., 2019). However, cleavage might not be required for others as unprocessed PROPEP3 was found to accumulate in the apoplast within 24 h after Pep treatment, pathogen challenge, and in response to cell wall damage (Yamada et al., 2016; Engelsdorf et al., 2018; Table 1). Downstream, Peps are perceived by the receptor-like kinases PEP receptor 1 and 2 (PEPR1 and PEPR2; Yamaguchi et al., 2006, 2010; Krol et al., 2010; Tang et al., 2015). Fluorescently labeled Pep1 travels locally in root tissue within a minute after external application and undergoes endocytosis when bound to PEPR1/2 (Ortiz-Morea et al., 2016). Recently, the Ca^2+^-permeable channel cyclic nucleotide gated channel 19 (CNGC19) was proposed to act downstream of Pep perception in generating Ca^2+^ fluxes during herbivory (Meena et al., 2019).

Peptidome approaches to identify native peptides directly from protein extracts allowed the identification of novel peptide DAMPs. A tomato pathogenesis related 1b (PR-1b) derived peptide identified from wounded and JA-treated plants forms the basis of a conserved family of CAPE peptides named after PR1b, which belongs to the cysteine-rich secretory proteins, antigen 5, and pathogenesis-related 1 proteins (CAP) superfamily (Chen et al., 2014; Chien et al., 2015). CAPE peptides operate during herbivore attack by activation of stress responsive genes, including proteinase inhibitors, and treatment with exogenous CAPE retards the growth of herbivores and confers resistance to *Pseudomonas syringae* pv. tomato DC3000 in tomato (Chen et al., 2014). In a recent peptidome approach, two interesting peptides were identified from developing *Arabidopsis* tracheary element cells (Escamez et al., 2019). Kratos and Bia (named after the children of the Styx river separating the worlds of the living and the dead in Greek mythology) decrease and enhance cell death during the incubation of leaf discs on the peptides, respectively (Escamez et al., 2019). While this hints at a novel role for Kratos in reducing wound-induced cell death, further investigation is needed.

#### Interactions Between DAMPs, HAMPs, and PAMPs

Herbivore associated molecular patterns (HAMPs) and pathogen associated molecular patterns (PAMPs) allow plants to perceive an attack from herbivores and pathogens, respectively, and interactions with responses to DAMPs have been described in the literature. Herbivory, for example, feeding by *Spodoptera* sp. caterpillars on Lima bean (*Phaseolus lunatus*) or *Medicago truncatula* or the application of HAMPs into mechanically inflicted wounds elicits conserved downstream signal amplification cascades (Duran-Flores and Heil, 2016). These cascades involve membrane depolarization, Ca^2+^ influxes, ROS formation, and the release of green leaf-volatiles (GLVs) within minutes, followed by MAPK phosphorylation and octadecanoid signaling cascades in the first hour following stress perception (Maffei et al., 2004, 2006; Arimura et al., 2008; Fürstenberg-Hägg et al., 2013; Schmelz, 2015). None of these responses are specific for a single type of herbivore or HAMP. Furthermore, in all cases of HAMP application, the leaves are mechanically damaged; hence the presence of DAMPs is unavoidable and the specific effects of DAMPs and HAMPs are difficult to be distinguished (Huffaker et al., 2013). Albeit a more artificial system, application of elicitors to suspension cell culture circumvents the unintended consequences of wounding and to disconnect the application of elicitors from the wound response (Shinya et al., 2018). Simultaneous application of *Oryza sativa* Pep3 and oral secretions from *Mythimna loreyi* has an additive effect on the production of ROS and MAPK activity and a synergistic effect on defense metabolite accumulation in comparison to separate application. This suggests that while DAMPs and HAMPs alone can trigger a defense response, perceiving both is critical for the strength of the induced plant defenses (Shinya et al., 2018).

A recent study provides a strong evidence for the positive interaction between wounding and PAMP recognition. Whereas applications of PAMPs do not or only weakly trigger immune-related gene expression in the *Arabidopsis* root, the co-incidence of accidental- or laser-induced damage highly amplifies this response as early as 4 h after wounding (Zhou et al., 2020). A localized and specific response is produced, as mostly close cells from underlying tissues, opposed to surrounding cells of the same tissue, respond strongly to the combination of PAMPs and damage. Wounding locally gates the expression of PAMP receptor kinases, and, thereby, immune responses to both beneficial or detrimental bacteria in roots. Co-application of the typical PAMP flg22 with DAMPS, including Pep1, eATP, cellobiose, OGs, or a cocktail thereof, however, does not induce immune-related gene expression to the extent as mechanical damage, suggesting that damage perception is more complex and likely involves other cues such as mechanical stress (Zhou et al., 2020).

Inceptin peptide is generated when cowpea (*Vigna unguiculata*) leaves are consumed by armyworm (*Spodoptera frugiperda*) larvae. Inceptin is produced by proteolysis of the cowpea chloroplastic ATP synthase γ-subunit (cATPC protein) in the insect gut and is then regurgitated back to the wound site (Schmelz et al., 2006). Inceptin stands in an intermediate position between HAMP and DAMP as conceptually speaking it is very similar, for example, to systemin, as it originates from a plant protein yet is different in the way that wounding alone does not trigger processing, and it requires a biotic attacker to process the peptide in order to trigger wound response (Duran-Flores and Heil, 2016). Inceptin is a disulfide-bridged peptide containing 11 amino acids. Exogenous treatment of cowpea with inceptin promotes the production of ethylene, SA and JA, and defense metabolite cinnamic acid, upregulates transcription of cowpea protease inhibitor, and enhances cowpea resistance to herbivory. Sequence alignments of cATPC proteins from multiple plant species demonstrate a high degree of conservation in the amino acid sequence related to the predicted inceptin peptides. However, inceptins are active elicitors of defense responses only in some Fabaceae (Schmelz et al., 2007; Li et al., 2020), suggesting that inceptin perception is a recent evolutionary event in plants. Recently, a leucine-rich repeat receptor-like kinase was found for inceptin in cowpea, being the first HAMP receptor to be reported and expanding the current knowledge of surface immune recognition to include herbivory (Steinbrenner et al., 2019).

### Keeping Your Friends Close: Local Damage Signaling by Ca^2+^, ROS, and Phosphorylation

Local wound signaling is defined as occurring typically a few cell layers away but, in terms of electrical signaling, can also relate to the whole wounded leaf (but not systemic leaves; see next section) and will depend on the severity of the wound. Receptor kinases, as mentioned in the previous sections, likely play an important role in perceiving a cocktail of DAMPs that is released in the immediate surrounding of wounds (Figure 1B). Ca^2+^ is a conserved second messenger involved in the initial signaling cascades of multiple physiological actions and in response to biotic and abiotic stresses (Kudla et al., 2010). Across scales of wounding, from single cell laser-mediated damage in roots to pin pricks and herbivory in leaves, cytosolic Ca^2+^ levels are the highest and remain elevated longer closest to the wound site (Beneloujaephajri et al., 2013; Costa et al., 2017; Behera et al., 2018; Nguyen et al., 2018; Toyota et al., 2018; Hander et al., 2019; Li T. et al., 2019; Marhavý et al., 2019). This observation also applies to other model species, for example, fruit fly (*Drosophila melanogaster*; Razzell et al., 2013; Shannon et al., 2017). Mechanical damaged cells themselves experience immediate and highest spikes in Ca^2+^ levels, likely because of passive influx of Ca^2+^ through perforated plasma membranes or coming from internal stores (Hander et al., 2019). Cytosolic Ca^2+^ peaks are associated with corresponding drops in cytosolic pH (Behera et al., 2018).

Calcium signaling relies on a set of channels, pumps, and effector Ca^2+^-binding proteins (De Vriese et al., 2018) for generation and readout of information in so-called Ca^2+^ signatures – cell-to-cell differences in calcium peak duration, intensity, and repetition – as observed during wounding (Figure 1C). Ca^2+^ signals can be inhibited by the application of typical extracellular chelators (e.g., EGTA and BAPTA) and inhibitors of Ca^2+^ channels (e.g., verapamil and GdCl3) at least in the cells neighboring the damaged cells (Beneloujaephajri et al., 2013; Hander et al., 2019; Marhavý et al., 2019). CNGC19 is the first known Ca^2+^-permeable channel that mediates propagation of cytosolic Ca^2+^ elevations in the vasculature of the local leaf (within a minute) during mechanical and herbivore damage (Meena et al., 2019). Loss-of-function *cncg19* mutants have a decreased production of JA, glucosinolates, and are more susceptible to herbivores (Meena et al., 2019). Free Ca^2+^ can bind to EF-hand motifs present in calmodulins, calcineurin B-like protein (CBL) and CBL-interacting protein kinase (CIPK), calcium-dependent protein kinases (CDPKs, also referred to as CPKs), and calmodulin-like proteins (CML). So far, autoinhibited Ca^2+^-ATPase isoform 8 (ACA8) is the only known Ca^2+^ pump involved in calcium signaling in the local wound response and is regulated by phosphorylation of a CBL1-CIPK9 complex (Costa et al., 2017). The Ca^2+^-binding protein, *CML42* is transcriptionally induced by *Spodoptera littoralis* feeding and application of insect oral secretions on *Arabidopsis* leaves but not by mechanical damage simulated by MecWorm (Mithöfer et al., 2005; Vadassery et al., 2012). Glucosinolate production is impaired in *cml42* mutants in the presence of herbivores. CML42 is responsible in part for the trichome branching formation, a structural defense against herbivores (Dobney et al., 2009), and in the negative modulation of JA-induced cytosolic Ca^2+^ elevations and JA signaling (Vadassery et al., 2012). On the contrary, *CML37* is induced both by insect herbivory and mechanical damage (MecWorm) and is a positive regulator of the defense response against herbivores, as JA accumulation and JA marker gene expression is impaired in *cml37* mutants upon herbivory (Scholz et al., 2014). The calmodulin binding protein IQD1 is induced by wounding and affects glucosinolate biosynthesis (Levy et al., 2005). From a collection of CPK mutants, *cpk3* and *cpk13* show lower levels of defense gene induction, independent of JA signaling, after wounding (Kanchiswamy et al., 2010). Interestingly, 30 min after mechanical or herbivore-induced damage, accumulation of intracellular Ca^2+^ at wound sites was significantly higher in *cpk3* than *cpk13* or wild type (Kanchiswamy et al., 2010).

Traditionally perceived as by-products of cellular metabolism, ROS have later been recognized to play active roles in stress signaling and to be essential for wound responses in plants and animals (Suzuki and Mittler, 2012). Hydrogen peroxide (H_2_O_2_) increases both at the injury site and systemically to reach a peak after 4–6 h, while superoxide (O_2_^−^) is believed to be transiently and locally generated within minutes after injury (Doke et al., 1991; Minibayeva et al., 2001; Orozco-Cárdenas et al., 2001). Next to providing structural roles in cell wall strengthening in response to mechanical damage (Bradley et al., 1992), ROS and especially the relatively more stable H_2_O_2_ can act as second messengers (Mignolet-Spruyt et al., 2016). Ca^2+^ and ROS accumulate locally following mechanical damage in the same cells, where Ca^2+^ accumulates in a few seconds and is required to initiate a subsequent longer-lasting increase of ROS (maximum at 10–12 min; Beneloujaephajri et al., 2013). Ca^2+^ and ROS intersect at the plasma membrane localized respiratory burst oxidase homolog (RBOH), which are plant homologs of NADPH oxidase (NOX) enzymes that contain Ca^2+^-binding EF-hand motifs. RBOHs function in propagation of systemic ROS waves (see next section), as well as local response, at least in *Arabidopsis* roots, leading to ethylene production (Marhavý et al., 2019). Similar to ROS, lesser-studied reactive nitrogen species (RNS), such as NO, accumulate locally between 15 min and 2 h and aid in wound healing by lignin and callose deposition (Huang et al., 2004; Corpas et al., 2008; Arasimowicz et al., 2009). ROS and RNS can affect the redox status of proteins, for example, through cysteine modifications, in biotic or abiotic stresses (Mhamdi, 2019). Cysteine oxidations are found in the enzymes 1-aminocyclopropane-1-carboxylic acid oxidase (ACO; ethylene) and 12-oxophytodienoic acid reductase 3 (OPR3; JA; McConnell et al., 2019; Pattyn et al., 2020), but the importance for wound response needs further investigation.

Classically, MAPK phosphorylation cascades, notably WIPK and SIPK in tobacco and homologs MPK3 and MPK6 in *Arabidopsis*, are activated at timescales between accumulation of Ca^2+^ (faster) and ROS (slower) with a maximum at 15 min after wounding (Seo et al., 1995, 1999; Usami et al., 1995; Bögre et al., 1997; Ichimura et al., 2000). Activation of upstream kinases include MEKK1 and MEK1 phosphorylating MKK2 and MPK4 in *Arabidopsis* (Matsuoka et al., 2002; Hadiarto et al., 2006), which can be reverted by the action of PP2C-type phosphatases (Schweighofer et al., 2007). Wound-induced MPK8 activity is detected within 10 min and is peculiar in the sense that both MKK3 phosphorylation and Ca^2+^-dependent calmodulin binding is required for full activation (Takahashi et al., 2011). Once activated, MPK8 controls the redox balance by negative regulation of *RBOHD* gene expression. Downstream of the wound-activated MKK4/MKK5-MPK3/MPK6 cascade and CPK5/CKP6 phosphorylation is the upregulation of ethylene biosynthesis genes and ethylene accumulation (Li et al., 2018). Intriguingly, next to the classical fast activation of MAPK cascades, a later activation controlled by JA-induced MAP3Ks expression and a cascade involving MKK3 phosphorylation of MPK1/2/7 can be observed with a maximum at 1 h after mechanical and herbivore-induced damage (Ortiz-Masia et al., 2007; Sözen et al., 2020).

Plasmodesmata are plasma membrane-lined pores that connect the cytoplasm of neighboring cells that allow cell-to-cell exchange of molecules, and the regulation thereof plays important roles in signaling of stresses, including pathogen defense and wounding (Jacobs et al., 2003; Cheval and Faulkner, 2018). Locally elevated levels of Ca^2+^ and ROS will lead to rapid closure of the plasmodesmata within seconds to minutes (Holdaway-Clarke et al., 2000; Cui and Lee, 2016; Xu et al., 2017). Deposition of callose, which is mostly Ca^2+^-dependent (Kauss et al., 1983; Leijon et al., 2018), will further “seal the deal” in prolonged closing of plasmodesmata and restricting access from the wound to intact tissues (Jacobs et al., 2003; Wu et al., 2018). In systemic signaling, plasmodesmata could be important for cell-to-cell movement of molecules or continuity of membranes and coupling of electrical signals (Cheval and Faulkner, 2018). Similarly, sieve plates of the phloem can be rapidly closed within minutes to prevent leakage of nutrients and assimilates by the deposition of callose (Mullendore et al., 2010). In Fabaceae, specialized proteinaceous structures called forisomes expand upon the binding of Ca^2+^ was released during wounding to block the sieve plate pores (Knoblauch and Van Bel, 1998). Interestingly, unidentified Ca^2+^-binding proteins in aphid (*Megoura viciae*) watery saliva, which they inject in the phloem, can chelate Ca^2+^ and leave sieve elements unblocked for uninterrupted aphid feeding in Fabaceae (Will et al., 2007). Cytosolic Ca^2+^ elevations during aphid feeding can be observed in species that lack forisomes, such as *Arabidopsis*, so Ca^2+^ chelation by aphid saliva is likely a more general phenomenon (Vincent et al., 2017).

### Systemic Wound Tides: Hydraulic Waves, Electric Torrents, and Ca^2+^ Fluxes

**Figure 2:**
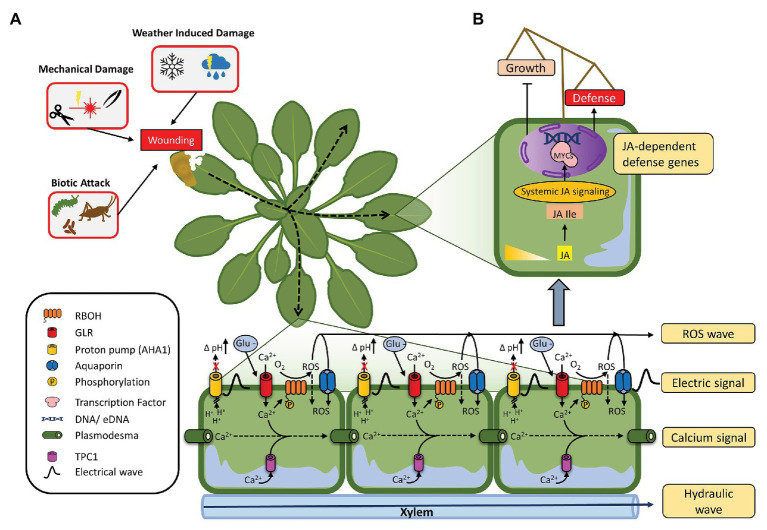
Schematic representation of systemic response to wounding. **(A)** Different origins of wounding, including biotic attack (herbivore and pathogens), mechanical damage (cutting and laser induced), and weather induced damage (freezing and hail). Depending on severity of the wound, propagation of systemic signals ensues. Local changes in membrane potentials, increases in cytosolic Ca^2+^, and ROS accumulation generate a wave that quickly spreads throughout the plant, in order to reach distant tissues. **(B)** Systemic-induced jasmonic acid (JA) continues to promote JA-dependent defense genes in distant tissues, leading to a systemic growth/defense trade-off to promote plant fitness.

More than a century ago, the existence of long-distance signals of unknown nature that is able to propagate signals throughout the plant and travel through the vascular bundle was already hypothesized (Burdon-Sanderson, 1873; Ricca, 1916; Stahlberg, 2006). In recent years, significant strides have been made in understanding these systemic signals (Davies, 2006; Stahlberg et al., 2006; Vodeneev et al., 2015; Farmer et al., 2020), which can be attributed mainly to (1) very rapid changes in hydraulic pressure and (2) slower propagation of electric, ROS, and Ca^2+^ signals, and enigmatic xylem-born chemical elicitor-dubbed Ricca’s factor (Ricca, 1916; Figure 2A). In parallel to vascular signaling, signals can be released from plants in volatile forms that may activate defense in the same plant’s distal parts or in other plants in the neighborhood (Kessler and Baldwin, 2001). Volatile signals are addressed in these recent reviews (Bouwmeester et al., 2019; Ninkovic et al., 2019).

Wounding can cause a direct loss of the water content of plants and in many occasions can disrupt the plant vasculature, which has a direct effect on the turgor pressure of plant epidermal cells (Malone and Stanković, 1991). Changes in the hydraulic components were proposed to be part of the systemic damage signal that takes advantage of the organ interconnectivity of the vasculature (Malone and Stanković, 1991; Boari and Malone, 1993). Another measure of hydraulic signals, found in common in different species including wheat, tomato, soybean, faba bean, and others, is a change in leaf thickness in neighboring leaves of a damaged leaf (Boari and Malone, 1993). Changes in turgor pressure and leaf thickness are likely caused by the retraction of water through the vascular system in a pressure wave that travels the rigid xylem vessels (Malone and Stanković, 1991; Stahlberg and Cosgrove, 1992, 1995). Although the results showed differences on the magnitude of the reaction across species and capacity of responsiveness, the data obtained for leaf thickness starts within seconds and peaks around 1–4 min, lasting about 1 h or longer. Hydraulic signals propagate at an estimated speed of 10–20 cm·s^−1^, meaning that rupture of the water continuity by wounding can have relatively direct repercussions on distant locations (Malone, 1992; Boari and Malone, 1993). At present, the study of hydraulic changes during wounding is rather unexplored, likely due to the absence of tools that allow efficient detection of changes on pressure over short periods of time at distant locations. A recent study detailed the use of a non-invasive method using optical methods that measures the changes of the diffraction patterns associated to stem displacement after flaming injury (Nožková et al., 2018).

Relatively better studied are the electrical signals, which are based on changes in the membrane potentials (depolarization or hyperpolarization followed by repolarization) and were recently reviewed in Farmer et al. (2020). At least four types of electrical signals elicited by damage are reported in the literature: wound potential, action potential, slow wave potential (also named variation potential), and systems potential, each displaying different characteristics (Davies, 2006; Stahlberg et al., 2006; Zimmermann et al., 2009, 2016; Farmer et al., 2020). Wound potential depolarization spreads locally around the damaged area (<40 mm or about the length of 200 epidermal cells in cucumber hypocotyls; Stahlberg et al., 2006). While probably sharing molecular mechanisms with systemic electrical signals, such as inhibition of P-type H+ pumps (Stahlberg et al., 2006), wound potentials are technically not considered as systemic signals. Action potentials, slow wave potentials, and systems potentials spread to distal parts of the plant with the main difference that slow wave potentials are driven by hydraulic or chemical changes, as they can travel across killed or poisoned areas (Stahlberg and Cosgrove, 1992). The slow part in slow wave potential reflects a delayed repolarization, and slow wave potentials are dampened in amplitude in more distal parts of the plant. On the other hand, action potentials are characterized by their all-or-none depolarization traveling without attenuation (Favre and Agosti, 2007; Cuin et al., 2018). Systems potentials are mainly different to the aforementioned signals in that they are hyperpolarized instead of depolarized (Zimmermann et al., 2009).

Earlier studies of electrical signals, similar to hydraulic signals, were mainly performed using harsh damaging treatments, such as flaming. More recently, subtle mechanical or herbivore induced wounds were also found to induce electrical signals, likely containing mixed forms of wound potentials, action potentials, slow wave potentials, and systems potentials (Salvador-Recatalà et al., 2014; Zimmermann et al., 2016). Observations of different electrical signals can be very heterogenous and depend on several factors including (1) severity of the damage, for example, flaming triggering strong hydraulic waves and slow wave potentials and herbivores triggering action potentials and systems potentials, (2) the readout method of choice, for example, stomata impaled-pierced, agar-pierced, or blindly pierced electrodes or aphids as living bioelectrodes, and (3) place of recording, which mainly relates to abundance and interplay of signals from multiple vascular strands (Salvador-Recatalà et al., 2014; Zimmermann et al., 2016).

Identification of clade 3 glutamate receptor-like (GLR) genes, *Arabidopsis* H + -ATPases (AHAs), and RBOHs that shape or propagate the systemic signals illustrate the intertwining of electrical signals with Ca^2+^ and ROS waves and their impact on the downstream activation of JA synthesis (Koo and Howe, 2009; Mousavi et al., 2013; Gilroy et al., 2016; Nguyen et al., 2018; Toyota et al., 2018; Farmer et al., 2020). Mousavi et al. (2013) identified two GLRs, homologs of mammalian ionotropic glutamate receptors (iGluRs), for which double homozygous mutants have reduced wound-induced systemic membrane depolarization, and changes in JA marker gene expression. While electric signals do not propagate to neighboring leaves in the *glr3.3 glr3.6* mutant, signals in the (local) wounded leaf are unaffected (Mousavi et al., 2013; Salvador-Recatalà et al., 2014; Salvador-Recatalà, 2016), leading to the conclusion that GLR3.3 and GLR3.6 are “gatekeepers” of systemic electric signals. Interestingly, loss-of-function of a third GLR, *glr3.5*, leads to systemic electric signals in non-neighboring leaves, where usually no signals are detected, indicating that GLR3.5 acts as an off-switch (Salvador-Recatalà, 2016). Whereas GLRs are involved in propagation of slow wave potentials, *in vitro* activation of CNGC19 by hyperpolarization hints at its involvement in systems potential propagation (Meena et al., 2019).

Wounds generated by mechanical damage results in the increase of apoplastic glutamate concentration ([Glu]apo) of ~50 mM within minutes at the damage site, suggesting that [Glu]apo can act as a DAMP (Toyota et al., 2018). Glutamate, among other amino acids, are specifically perceived in plants through GLRs (Qi et al., 2006; Toyota et al., 2018; Alfieri et al., 2020; Shao et al., 2020). GLRs are calcium-permeable channels and thereby mediate the influx of cytosolic Ca^2+^ within seconds after the damage (Vincill et al., 2012; Mousavi et al., 2013; Nguyen et al., 2018; Toyota et al., 2018). Similar to slow wave potentials, systemic Ca^2+^ waves are observed following wounding (Kiep et al., 2015) and did not spread to neighboring intact leaves in the *glr3.3 glr3.6* mutant (Toyota et al., 2018), showing that electrical and Ca^2+^ signals are closely interacting through GLRs (Nguyen et al., 2018). In *Arabidopsis* systemic leaves, slow wave potentials seem to precede peak Ca^2+^ signals (Nguyen et al., 2018).

Slow wave potentials travel through the vasculature toward the center of the rosette and then disperse away from the apex into a restricted number of parastichy leaves to initiate distal JA accumulation and signaling (Mousavi et al., 2013; Nguyen et al., 2018; Farmer et al., 2020; Figure 2B). The measured speed of leaf-to-leaf electrical signal was observed in the ~2 cm/min range, which is concordant with estimates of signal speeds of JA accumulation measured in leaf-to-leaf wounding studies (Chauvin et al., 2013; Mousavi et al., 2013). The signal spreads across tissues by GLRs through the phloem and xylem vascular tissues, especially when major veins are damaged (Salvador-Recatalà et al., 2014; Nguyen et al., 2018; Toyota et al., 2018). Minutes following slow wave potentials, JA is locally and systemically synthesized leading to the activation of the transcriptional JA responses (Mousavi et al., 2013). Proton pumps were long expected to take part in the return of membrane potential back to its initial status (repolarization), but the genetic evidence was lacking (Stahlberg and Cosgrove, 1996; Fleurat-Lessard et al., 1997). Kumari et al. (2019) recently found that repolarization in the *Arabidopsis* proton pump H + -ATPASE 1 (AHA1) deficient plants took longer compared to wild type, indicating a role for AHA1 in resetting the plant for sensing new stimuli. Additionally, *aha1* mutants have higher total JA accumulation and JAZ10 expression and reduced levels of herbivory (Kumari et al., 2019), which is the opposite in *glr3.3 glr3.6* plants (Mousavi et al., 2013; Nguyen et al., 2018). Recently as well, Shao et al. (2020) provided evidence that higher pH, such as during wound-induced apoplast alkalization, greatly enhances the binding of glutamate to GLR3.3 and GRL3.6. They further confirmed the effect of AHA1 on slow wave potentials. Taking in consideration theoretical models and experimental work that predict chemical agents transported by xylem mass flow or sheer-enhanced dispersion propagate slow wave potentials, as opposed to pressure waves (too fast) or chemical diffusion (too slow; Vodeneev et al., 2012; Evans and Morris, 2017; Blyth and Morris, 2019), [Glu]apo might well be (part of) the ideal candidate(s) for Ricca’s long-sought chemical factor(s) that propagate the slow wave potential (Ricca, 1916). Sudden changes in the negative and positive pressure of xylem and phloem, respectively, followed by osmotic re-equilibration, might help to pull in [Glu]apo or other chemical elicitors in the vasculature (Farmer et al., 2020).

In parallel with systemic electrical signals, Ca^2+^ and ROS waves are induced by wounding, among other stresses, and depend on RBOHs (Miller et al., 2009; Choi W.-G. et al., 2014; Kiep et al., 2015; Evans et al., 2016; Choi et al., 2017; Toyota et al., 2018). In systemic tissues, mechanical damage and H_2_O_2_ inducible gene expression overlap considerably more than any other purportedly ROS-induced transcripts, including O_2_^−^ and singlet oxygen (Miller et al., 2009). One of these H_2_O_2_-inducible genes is zinc finger of *Arabidopsis thaliana*12 (ZAT12). *ZAT12* expression, using luciferase reporter lines, is induced strongly within 10 min after wounding in the local leaf, while it spreads systemically at 8.4 cm/min to full expression within the hour and both are impaired in an *rbohd* mutant (Miller et al., 2009). New ways of visualizing ROS will improve the further study of systemic signaling in species other than *Arabidopsis*, including crops (Fichman et al., 2019; Lew et al., 2020). ROS waves can be inhibited by the Ca^2+^ channel blocker lanthanum (La3^+^; Miller et al., 2009). Next to the N-terminal Ca^2+^-binding EF-hand motif (Suzuki et al., 2011), RBOHD activity is regulated through phosphorylation at its N-terminus by the calcium dependent kinase CPK5 upon elicitation with flg22, a bacterial flagellin peptide and elicitor of innate immunity (Suzuki et al., 2011; Dubiella et al., 2013). Wound-induced Ca^2+^ waves are suppressed in loss-of-function mutants of the vacuolar cation channel two pore channel 1 (TPC1), whereas local Ca^2+^ elevation was largely unaffected (Kiep et al., 2015). RBOHD can interact with different partners involved in immune response such as the receptor kinases EFR and FLS2, and botrytis-induced kinase1 (BIK1; Laluk et al., 2011; Kadota et al., 2014). Furthermore, cysteine rich receptor-like kinase 2 (CRK2) controls flg22-induced H_2_O_2_ production through direct interaction with RBOHD and phosphorylation of its cytosolic C-terminus (Kimura et al., 2020). Whether these interactions are important for systemic wound signaling warrants investigation. A unifying concept of molecular mechanisms underpinning wound-induced systemic signals is within reach (Gilroy et al., 2016; Farmer et al., 2020) but will require the discovery of additional genetic players.

## Wound-Induced Hormone Signaling

Upon wounding, several hormones, including JA, ethylene, ABA, auxin, and their respective cross-talks, are indispensable for damage perception and eliciting key downstream responses.

### First on the Scene: Jasmonic Acid Signaling

Jasmonic acid is a phytohormone involved in many aspects of plant stress responses and development. Probably the most renowned is the regulation of mechanical wounding and immune responses against herbivores or necrotrophic pathogens, which trigger the biosynthesis of JA and of its bioactive form jasmonoyl-l-isoleucine (JA-Ile) not only at the damage site but also systemically in unharmed tissues (Glauser et al., 2008; Koo and Howe, 2009; Goossens et al., 2016). JA biosynthesis begins with release of α-linolenic acid from chloroplast membrane phospholipids, which is then converted into cis-(+)12-oxo-phytodienoic acid (OPDA) through the sequential action of chloroplast-located enzymes, such as the 13-lipoxygenases (13-LOX; Wasternack and Feussner, 2018). OPDA is then exported from the chloroplast by JASSY, a protein localized to the outer chloroplast envelope (Guan et al., 2019), and transported into the peroxisomes, presumably by the ABC transporter Comatose (AtABCD1/CTS) and acyl-CoA-binding protein 6 (ACBP6; Theodoulou et al., 2005; Ye et al., 2016). Once in the peroxisome, OPDA is reduced by OPDA reductases 2 and 3 (OPR2 and OPR3) and subsequently oxidized through two distinct pathways to form JA (Schaller and Stintzi, 2009; Chini et al., 2018). The bioactive molecule JA-Ile is synthetized by the JA resistant 1 (JAR1) enzyme and transported to the nucleus within minutes after plant damage (Suza and Staswick, 2008). Nuclear transport of JA-Ile is mediated by the jasmonate transporter 1 (JAT1), a member of the ABC transporter family known to transport small molecules such as auxins, abscisic acid, or strigolactones (Li et al., 2017). In the nucleus, JA-Ile is perceived by a co-receptor complex composed of the JA ZIM-domain (JAZ) repressor proteins and the coronatine insensitive 1 (COI1) F-box protein that associates with CUL1, Rbx1, and the Skp1-like proteins ASK1 and ASK2 to assemble the SCF-COI1 ubiquitin-ligase complex (Thines et al., 2007; Fonseca et al., 2009; Sheard et al., 2010; Williams et al., 2019). Hormone perception requires a JAZ degron that bridges COI1 to JA-Ile (Sheard et al., 2010). In addition, inositol pentakisphosphate (InsP_5_) was identified as a critical structural component of the receptor complex (Sheard et al., 2010). Plants with increased InsP5 showed accentuated wounding responses, suggesting that InsP5 contributes to the assembly and function of the SCF-COI1 complex (Mosblech et al., 2011). Following JA-Ile binding, the SCF-COI1 complex ubiquitinates the JAZs, which targets them for proteasomal degradation. Thereby, several transcription factors (TFs), such as the MYCs that are otherwise bound by the JAZ proteins, are released and can activate the JA response (Chini et al., 2007; Fonseca et al., 2009; Goossens et al., 2016). JA-Ile perception and signaling leads to the systemic alteration of a growth-defense balance to promote plant fitness (Wasternack and Feussner, 2018). One of the most characteristic features of JA is the transcriptional reprograming of a wide array of enzymes leading to production of specialized metabolites, including terpenes, glucosinolates, phenolics, or alkaloids (Pauwels et al., 2008; Hickman et al., 2017; Zander et al., 2020).

On the contrary, JA represses signaling pathways that lead to plant growth to reallocate resources toward defense (Hou et al., 2010; Major et al., 2017; Guo et al., 2018). It was shown that a growth penalty is restored to different extents in moderate (*jazQ*) or in severe (*jazD*) JAZ depleted mutants by the introgression of a *phytochrome B* (*phyB*) mutation, which was explained by the fact that JA and phyB transcriptional networks are uncoupled (Campos et al., 2016; Major et al., 2020). Interestingly, these findings show that the JA regulated growth-defense trade-off is not merely directed by the need of relocating metabolic resources, which opens interesting leads for plant improvement for agricultural or industrial purposes. Because of the importance in tuning the growth-defense balance, JA and growth promoting pathways are cross-regulated through different pathways in response to changing environments. DELLA proteins are plant growth repressors whose degradation is promoted by gibberellins (Davière and Achard, 2016). DELLAs have been reported to interact with JAZs to thereby compete with MYC2 and, thus, modulate JA responses (Hou et al., 2010; Wild et al., 2012; Leone et al., 2014). However, the importance of DELLAs in the cross-regulation of the JA pathway has recently been challenged by a study that shows no major role of DELLAs in restricting shoot growth of jaz mutants (Major et al., 2020). Wounding dramatically modifies the growth-to-defense balance, resulting in stunted vegetative growth effects being directly linked to the activation of JA synthesis (Yan et al., 2007).

A key function of JAs produced in damaged organs is to travel systemically across tissues in order to reprogram future growth and optimize plant defense strategies (Huot et al., 2014; Guo et al., 2018; Ballaré and Austin, 2019). Upon damage, plants tightly regulate biosynthesis, transport, and catabolism of JAs (Browse, 2009; Chini et al., 2016; Howe et al., 2018; Fernández-Milmanda et al., 2020; Yang et al., 2020). JA biosynthesis in *Arabidopsis* depends on LOX2, LOX3, LOX4, and LOX6. Each of these LOXs contribute in a different way to regulate JA biosynthesis and transport upon wounding (Chauvin et al., 2013, 2016; Grebner et al., 2013; Yang et al., 2020). *LOX2* is expressed throughout soft aerial tissues, whereas *LOX3, LOX4*, and *LOX6* are expressed in the phloem and xylem of leaves (Chauvin et al., 2013, 2016). *LOX2* is highly induced in the close vicinity of wounds in cotyledons and is necessary to ensure leaf to root axial JA transport (Gasperini et al., 2015). Upon wounding, LOX6 participates in the radial export of JAs from the leaf vasculature to the blade (Gasperini et al., 2015). It was recently suggested that LOX3 and LOX4 repress leaf growth upon wounding by acting on stem cell populations that determine the rate of leaf primordia development (Yang et al., 2020). Furthermore, the activity of LOX3 and LOX4 in leaf growth is related to the vacuolar cation channel TPC1 through a mechanism that remains unclear (Bonaventure et al., 2007; Yang et al., 2020).

The aforementioned studies together with the discovery of GLR-aided electrical signaling reveal that wounded leaves rely on at least two kinds of JA-dependent mechanisms to alert distal organs, being different whether the signal translates from leaf-to-leaf or from leaf-to-root (Mousavi et al., 2013; Gasperini et al., 2015; Schulze et al., 2019). Shoot wounding not only activates electrical signals but also triggers relocation of JA-Ile precursors, tentatively OPDA, OPC-4, OPC-6, OPC-8, and JA from wounded shoots toward undamaged roots (Schulze et al., 2019). Mobile OPDA and its derivatives activate JA signaling through their conversion into JA-Ile at the distal sites, and while leaf-to-leaf signaling relies on electrical and hormone translocation mechanisms, and leaf-to-root signaling seems to exclusively rely on hormone (precursor) translocation (Schulze et al., 2019). In complement to these studies, the development of the fluorescent biosensor Jas9-VENUS allowed visualization of the dynamic distribution of JA-Ile in wounded plants (Larrieu et al., 2015). Cotyledon wounding generated a distal increase of JA-Ile through vascular tissues of the root following two distinct temporal dynamics. The first phase started with a rapid increase of distant JA-Ile propagating at a speed <1 cm/min, few minutes after wounding, then a second slower phase that started 30 min and lasted for at least 90 min (Larrieu et al., 2015). The nature behind these phases needs further investigation to be conciliated with latter results, suggesting that leaf-to-root signaling exclusively relies on hormone translocation, which is likely a slower process than the initial observed phase.

Although glutamate was characterized as triggering rapid slow wave potentials resulting in the activation of the JA pathway, little is known about additional DAMPs triggering distant and/or local JA signaling. A large set of cell wall-related DAMPs have been characterized for triggering wound responses; however, despite the fact that JA is one of the most well-characterized phytohormonal pathways activating wound responses, mechanisms clearly connecting cell wall perception to JA are missing (Mielke and Gasperini, 2019; Bacete and Hamann, 2020). Exogenous application of cell wall degrading enzymes or the cell wall fragments OGs (DP10–DP15) or xyloglucans (Xh) results in the activation of JA signaling (de Azevedo Souza et al., 2017; Claverie et al., 2018; Engelsdorf et al., 2018). Xh elicited resistance against the necrotrophic pathogen *Botrytis cinerea* was abolished in JA biosynthesis (*dde2*) and signaling (*coi1-40*) mutants, suggesting the specific activation of the JA pathway by Xh (Claverie et al., 2018). In *Nicotiana attenuata*, the combination of wounding with the fatty acid conjugates N-linolenoyl-l-Gln, N-linolenoyl-l-Glu, N-linoleoyl-l-Gln, and N-linoleoyl-l-Glu strongly activated JA biosynthesis and subsequent herbivore defense responses (Wu et al., 2007). Future studies should address how cell wall disruption leads to local JA signals and if they connect to systemic responses. In this respect, *Arabidopsis* mutants of the xylem-specific cellulose synthases, irregular xylem 3 and 5 (*irx3* and *irx5*), severely affect the shape and speed of slow wave potentials; however, *JAZ10* expression in systemic leaves, as a measure of JA signaling, is only slightly affected (Kurenda et al., 2019).

Likewise, the molecular events operating downstream of the Ca^2+^ influx, preceding the rapid biosynthesis and redistribution of JAs are hardly understood. Phosphorylation is postulated to be one of the major cellular modes of action for translating defined Ca^2+^ signatures into specific downstream reactions (Dodd et al., 2010; Yip Delormel and Boudsocq, 2019). Several existing lines of evidence point to the importance of Ca^2+^/phosphorylation for JA signaling in the context of wound responses. Furthermore, Ca^2+^ signaling has been repeatedly hypothesized as a mechanism preceding JA signaling, which suggests that Ca^2+^ signals may not only relate to GLRs but also to other alternative pathways activating JA signaling (Kenton et al., 1999; Bonaventure et al., 2007; Scholz et al., 2014; Lenglet et al., 2017). The JA-associated VQ motif 1 (JAV1) protein associates in a complex with JAZ8-WRKY51 to represses the expression of JA biosynthesis genes. Wounding or insect attack activate a Ca^2+^Calmodulin dependent pathway that phosphorylates JAV1, leading to its degradation to thereby activating transcription of JA biosynthesis genes (Yan et al., 2018). Beyond the potential importance of phosphorylation for Ca^2+^ induced JA biosynthesis, it was recently shown that wounding triggers JA signaling in the stomata through the activity of the Ca^2+^ receptor kinase complex CBL1-CIPK5 (Förster et al., 2019). Furthermore, a recent study showed that the rice homolog of brassinosteroid insensitive 2 (BIN2), OsGSK2 kinase, phosphorylates OsJAZ4 to promote its degradation in a COI1-dependent manner, thereby posing a new mechanism of growth-defense regulation (He et al., 2020). Additionally, wound-activated MAPK signaling mechanisms have been reported to regulate the JA pathway (Wu et al., 2007; De Boer et al., 2011). WIPK and SIPK regulate wound responses including JA biosynthesis in Solanaceae species. In *N. attenuata*, leaf wounding together with the herbivore oral secretion treatment elicits strong SIPK and WIPK activities resulting in the biosynthesis of JA, SA, and JA-Ile/JA-Leu conjugates and ethylene biosynthesis. The SIPK and WIPK activate the transcription of defense related genes in both wounded and unwounded regions of the local leaf but not in systemic adjacent leaves (Wu et al., 2007). In tobacco, the JA-factor stimulating MAPKK1 (JAM1) protein regulates transactivating activities of the ORC1 and MYC transcription factors in a JA dependent manner (De Boer et al., 2011). Altogether, this evidence underscores that phosphorylation is an important post-translational modification in the regulation of plant wound responses and JA signaling; however, to date, these mechanisms have only been explored to a limited extent.

### Rather Late Than Never: Accumulation of Ethylene, ABA, and Auxin During Wound Response

Ethylene has many roles in plant development and stress response (Pattyn et al., 2020), including fruit ripening, where inhibition is a critical target for improved storage (shelf-life) of fruit and vegetables post-harvest (Barry and Giovannoni, 2007; Saltveit, 2016). Wound-induced ethylene accumulation is thought to proceed *via* transcriptional upregulation of its rate-limiting biosynthetic enzyme 1-aminocyclopropane-l-carboxylate (ACC) synthase (ACS) resulting in a lag-time of 20–30 min before the first accumulation of ethylene and a peak within hours after wounding (Boller and Kende, 1980; Kato et al., 2000; Marhavý et al., 2019). Ethylene production depends on both ROS and Ca^2+^ increases (Marhavý et al., 2019) and is transduced by MAPK and CDPK-dependent phosphorylation for activation of *ACS* gene expression locally at wound sites (Wu et al., 2007; Li et al., 2018; Sözen et al., 2020). Possible involvement of DAMPs cannot be ruled out, as Peps induce the accumulation of ethylene within 5 h after exogenous peptide application (Bartels et al., 2013). Furthermore, electrical signaling might lead to systemic increases of ethylene production in distal leaves (Dziubinska et al., 2003; Tran et al., 2018; Farmer et al., 2020). In the young root meristem, JA has been shown to be involved in transmitting the single cell damage signal (Zhou et al., 2019), whereas in older non-dividing root cells, a predominant role for ethylene has been demonstrated (Marhavý et al., 2019). Here, death of a single cell, through laser ablation or during the early stages of nematode infection, causes a distinct ethylene-dominated response (Marhavý et al., 2019).

Abscisic acid (ABA) accumulation is doubled within 24 h after wounding and induces, among other cues, the expression of the *proteinase inhibitor II* gene in potato and tomato (Pēna-Cortes et al., 1989; Peña-Cortés et al., 1995; Dammann et al., 1997). Arguably, ABA is best known for its role in drought-induced stomatal closure (Cardoso and McAdam, 2019). Therefore, it should come as no surprise that ABA accumulation likely depends on the level of humidity during wounding. As a case in point, *Arabidopsis* plants accumulate ROS normally and develop wound induced resistance (WIR) to the fungus *Botrytis cinerea* in high humidity (L’Haridon et al., 2011). However, keeping plants 1.5 h in dry conditions after wounding, reduces ROS, WIR, and callose accumulation, which is linked to enhanced accumulation of ABA and is reversed in ABA biosynthetic enzyme deficient mutants *aba2* and *aba3* (L’Haridon et al., 2011). In this study, ABA biosynthesis genes are induced 15 min after wounding only in the dry condition. Probably, differences in experimental set-ups, therefore, fail to detect changes in wound-induced ABA accumulation and gene transcription (Ikeuchi et al., 2017). Interestingly, an application of exogenous ABA leads to enhanced local cell death surrounding wound sites in *Arabidopsis*, and the transcription factor botrytis sensitive1/MYB108 (BOS1/MYB108) is a negative regulator of this ABA-dependent cell death (Cui et al., 2013). Mutant *bos1* plants display runaway cell death after wounding, which interacts with ABA, cuticle permeability, and resistance to *B. cinerea* (Cui et al., 2013, 2016, 2019).

Accumulation of auxin at wound sites mainly has a role in the subsequent repair process that bridges or protects wounds and regeneration of lost tissue. Tissue reunion following incision or upon grafting requires reactivation of cell division, not so much to generate callus, but rather to bridge the cut and allow reconnection of the vascular tissue. Upon incision of the inflorescence stem, the NAC-type transcription factor NAC071 and ERF113 are activated in order to assist in the reunion process (Asahina et al., 2011). On the one hand, ERF113, an AP2/ERF-type transcription factor, is rapidly induced within 1 day following incision at the bottom part of the cut site in a JA-dependent manner. On the other hand, NAC071 is induced in the top part of the incision between 1 and 3 days as a result of auxin accumulation, and both TFs execute different functions in the reunion process (Asahina et al., 2011). Auxin response during grafting is symmetric between top and bottom of the adjoined graft junction and occurs within 12 h, consistent with earlier reports of auxin-induced transcription at 1–3 days after cutting (Yin et al., 2012; Melnyk et al., 2015, 2018; Matsuoka et al., 2016). Upon full excision of the leaf between the blade and petiole, callus is generated very locally at the cut site and an adventitious root can sprout within 8 days following excision (Liu et al., 2014). Auxin accumulates within a day at the wound site and directly activates expression of the *WUSCHEL related homeobox 11* (*WOX11*) transcription factor, which works redundantly with WOX12 to enable the transition of the local cambium cells to root founder cells within 4 days following the cut (Liu et al., 2014; Hu and Xu, 2016). Accumulating evidence from recent publications on root regeneration emphasizes the importance of auxin during the replenishment of a single cell, a cluster of damaged root cells, or even regeneration of a complete *de novo* root tip (Canher et al., 2020; Hoermayer et al., 2020; Matosevich et al., 2020). Depending on the severity and type of damage, the mode of action that allows for sufficient auxin accumulation in order to facilitate the regeneration process varies. Upon death of a single cell, for example, following laser ablation, a strictly localized auxin signaling, independent of biosynthesis or active transport, coordinates the wounding response (Hoermayer et al., 2020). Upon death of a group of vascular stem cells, for example, by bleomycin-induced DNA damage, the natural auxin flow is disrupted through downregulation of auxin transporters, resulting in an auxin redistribution, much alike rocks in a stream. However, similar to single cell death, no auxin biosynthesis could be observed during the recovery from vascular stem cell death (Canher et al., 2020). However, following full root tip excision, YUCCA9-dependent auxin biosynthesis was found to be indispensable to allow regeneration of a *de novo* tip (Matosevich et al., 2020). Among the key regeneration-related and wound-responsive transcription factors, several members of the AP2/ERF-type of transcription factors can be found, including ERF115, wound-induced dedifferentiation 1 (WIND1) and several plethora (PLT) members (Delessert et al., 2004; Iwase et al., 2011; Ikeuchi et al., 2013; Heyman et al., 2016). Although originally identified as a rate-limiting factor controlling stress-induced quiescent cell divisions, *ERF115* represents an important wound-responsive gene (Heyman et al., 2013, 2016). Being the death of a single cell, stem cell damage or even removal of the entire root tip, *ERF115* expression is instantly activated within 1–2 h in the adjacent cells and plays a key role in stimulating these cells to activate the cell division program (Heyman et al., 2016; Zhang et al., 2016). Although not being the initial trigger, auxin is required to maintain *ERF115* expression following tissue damage (Canher et al., 2020; Hoermayer et al., 2020), leaving the question open about the initial trigger activating this key regeneration granting factor following wounding.

## Future Perspectives

The field has come a long way since the first observations and descriptions of plant wound response more than a century ago (Burdon-Sanderson, 1873; Ricca, 1916; Bloch, 1941; Lipetz, 1970). Notwithstanding detailed molecular knowledge gathered in the last decades on several aspects, major areas of study are still largely unexplored. Keeping in the spatiotemporal spirit of the review, some of these areas can be defined from local to systemic and fast to slow.

What is the fate of damaged cells in the wound and are they actively involved in determining the outcome of the wound response? This is exemplified by the activation of metacaspases and maturation of Peps in damaged cells (Hander et al., 2019), which shows that “post-mortem” cells can still be active (Bollhöner et al., 2013). Furthermore, what are the chain of events that proceed in the dying cells bordering the damaged cells as observed in leafs (Cui et al., 2013; Iakimova and Woltering, 2018), is there a point of no return and how does it change the wound response in the neighboring tissue? While more DAMPs are being discovered, possible mechanisms that are in place to avoid unwanted or exaggerated wound response by maturation, possible controlled release, and turnover of DAMPs become important. Furthermore, are there different dynamics of DAMP release, for example, fast elevation of eATP and [Glu]apo (Song et al., 2006; Toyota et al., 2018), compared to potential slow release of OGs due to upregulation of polygalacturonases after wounding (Bergey et al., 1999)? Some studies have detailed the release of DAMPs after wounding (Table 1) or extrapolate from studies in animal model species. However, most DAMPs in plants have not been directly measured in the apoplast or vasculature during wounding, while there is an abundance of indirect measurements (e.g., exogenous application). To fully understand the dynamics of DAMP release and its impact on wound response, direct measurements are needed in the future.

In this review, we had to limit ourselves to reports dealing with wounding. Certainly, molecular components that are increasingly found in other abiotic or biotic stresses for local and systemic signaling likely play roles as well in wounding. As an illustration, ROS-mediated activation of Ca^2+^ channels by the receptor kinase HPCA1 (Wu et al., 2020) or mechanisms that have been described for systemic signaling by stresses other than wounding (Gilroy et al., 2016; Szechyńska-Hebda et al., 2017; Farmer et al., 2020). Local implications and responses of cells to wounding change in different tissues. For example, mesophyll cells are differently connected as xylem or phloem cells that form conduits. Disruption of tissue integrity will therefore have different repercussions, which is obvious in the slow-down of electrical, Ca^2+^, and ROS waves when they move from vasculature to inner tissues (Salvador-Recatalà et al., 2014; Evans et al., 2016; Toyota et al., 2018). Similarly, non-dividing full-grown cells and tissues will have different needs than expanding tissues and meristem cells, which are more plastic and essential to replace. The differences in these tissue-specific wound responses are only starting to be addressed (Hoermayer and Friml, 2019; Li T. et al., 2019; Marhava et al., 2019; Marhavý et al., 2019; Zhou et al., 2019).

Although such complex problems are difficult to predict (Lehmann et al., 2020), detailed knowledge on plant wound response will become even more needed as weather- and herbivore-induced damage is projected to increase with climate change (Deutsch et al., 2018). Development of new techniques for investigating wound response, such as MecWorm (Mithöfer et al., 2005) and SpitWorm that adds oral secretion to simulated herbivore-induced damage (Li G. et al., 2019), or laser-mediated wounding (Hoermayer and Friml, 2019; Marhavý et al., 2019) will help advance the field. Application of this newfound knowledge has the ability to improve grafting, regeneration, and crop production (Santamaria et al., 2013; Si et al., 2018; Coppola et al., 2019; Notaguchi et al., 2020; Zhang and Gleason, 2020).

## Author Contributions

IV-M, DD-F, and AF-F made the table and figures. IV-M and DD-F wrote the part on DAMPS. AF-F and SS wrote the part on local and systemic wound signaling. AR, JH, and SS wrote the part on hormones. All authors made comments on the manuscript, which were integrated by SS. All authors contributed to the article and approved the submitted version.

### Conflict of Interest

The authors declare that the research was conducted in the absence of any commercial or financial relationships that could be construed as a potential conflict of interest.

## References

[ref1] Agrawal A. A. (1998). Induced responses to herbivory and increased plant performance. Science 279, 1201–1202. 10.1126/science.279.5354.1201, PMID: 9469809

[ref2] Albert M. (2013). Peptides as triggers of plant defence. J. Exp. Bot. 64, 5269–5279. 10.1093/jxb/ert275, PMID: 24014869

[ref3] Alfieri A. Doccula F. G. Pederzoli R. Grenzi M. Bonza M. C. Luoni L.. (2020). The structural bases for agonist diversity in an *Arabidopsis thaliana* glutamate receptor-like channel. Proc. Natl. Acad. Sci. U. S. A. 117, 752–760. 10.1073/pnas.1905142117, PMID: 31871183 PMC6955363

[ref4] Arasimowicz M. Floryszak-Wieczorek J. Milczarek G. Jelonek T. (2009). Nitric oxide, induced by wounding, mediates redox regulation in pelargonium leaves. Plant Biol. 11, 650–663. 10.1111/j.1438-8677.2008.00164.x, PMID: 19689772

[ref5] Arimura G. I. Garms S. Maffei M. Bossi S. Schulze B. Leitner M.. (2008). Herbivore-induced terpenoid emission in *Medicago truncatula*: concerted action of jasmonate, ethylene and calcium signaling. Planta 227, 453–464. 10.1007/s00425-007-0631-y, PMID: 17924138 PMC2756395

[ref6] Asahina M. Azuma K. Pitaksaringkarn W. Yamazaki T. Mitsuda N. Ohme-Takagi M.. (2011). Spatially selective hormonal control of RAP2.6L and ANAC071 transcription factors involved in tissue reunion in *Arabidopsis*. Proc. Natl. Acad. Sci. U. S. A. 108, 16128–16132. 10.1073/pnas.1110443108, PMID: 21911380 PMC3179063

[ref7] Bacete L. Hamann T. (2020). The role of mechanoperception in plant cell wall integrity maintenance. Plants 9:574. 10.3390/plants9050574, PMID: 32369932 PMC7285163

[ref8] Ballaré C. L. Austin A. T. (2019). Recalculating growth and defense strategies under competition: key roles of photoreceptors and jasmonates. J. Exp. Bot. 70, 3425–3436. 10.1093/jxb/erz237, PMID: 31099390

[ref9] Barbero F. Guglielmotto M. Capuzzo A. Maffei M. E. (2016). Extracellular self-DNA (esDNA), but not heterologous plant or insect DNA (etDNA), induces plasma membrane depolarization and calcium signaling in Lima bean (*Phaseolus lunatus*) and maize (*Zea mays*). Int. J. Mol. Sci. 17:1659. 10.3390/ijms17101659, PMID: 27690017 PMC5085692

[ref10] Barry C. S. Giovannoni J. J. (2007). Ethylene and fruit ripening. J. Plant Growth Regul. 26, 143–159. 10.1007/s00344-007-9002-y

[ref11] Bartels S. Boller T. (2015). Quo vadis, Pep? Plant elicitor peptides at the crossroads of immunity, stress, and development. J. Exp. Bot. 66, 5183–5193. 10.1093/jxb/erv180, PMID: 25911744

[ref12] Bartels S. Lori M. Mbengue M. van Verk M. Klauser D. Hander T.. (2013). The family of Peps and their precursors in *Arabidopsis*: differential expression and localization but similar induction of pattern-triggered immune responses. J. Exp. Bot. 64, 5309–5321. 10.1093/jxb/ert330, PMID: 24151300

[ref13] Basu S. Varsani S. Louis J. (2018). Altering plant defenses: herbivore-associated molecular patterns and effector arsenal of chewing herbivores. Mol. Plant-Microbe Interact. 31, 13–21. 10.1094/MPMI-07-17-0183-FI, PMID: 28840787

[ref14] Becerra-Moreno A. Redondo-Gil M. Benavides J. Nair V. Cisneros-Zevallos L. Jacobo-Velázquez D. A. (2015). Combined effect of water loss and wounding stress on gene activation of metabolic pathways associated with phenolic biosynthesis in carrot. Front. Plant Sci. 6:837. 10.3389/fpls.2015.00837, PMID: 26528305 PMC4606068

[ref15] Behera S. Xu Z. Luoni L. Bonza M. C. Doccula F. G. De Michelis M. I.. (2018). Cellular Ca^2+^ signals generate defined pH signatures in plants. Plant Cell 30, 2704–2719. 10.1105/tpc.18.00655, PMID: 30377237 PMC6305977

[ref16] Bellincampi D. Cervone F. Lionetti V. (2014). Plant cell wall dynamics and wall-related susceptibility in plant-pathogen interactions. Front. Plant Sci. 5:228. 10.3389/fpls.2014.00228, PMID: 24904623 PMC4036129

[ref17] Bellincampi D. Dipierro N. Salvi G. Cervone F. De Lorenzo G. (2000). Extracellular H_2_O_2_ induced by oligogalacturonides is not involved in the inhibition of the auxin-regulated *rolB* gene expression in tobacco leaf explants. Plant Physiol. 122, 1379–1385. 10.1104/pp.122.4.1379, PMID: 10759534 PMC58973

[ref18] Beloshistov R. E. Dreizler K. Galiullina R. A. Tuzhikov A. I. Serebryakova M. V. Reichardt S.. (2018). Phytaspase-mediated precursor processing and maturation of the wound hormone systemin. New Phytol. 218, 1167–1178. 10.1111/nph.14568, PMID: 28407256

[ref19] Benedetti M. Pontiggia D. Raggi S. Cheng Z. Scaloni F. Ferrari S.. (2015). Plant immunity triggered by engineered *in vivo* release of oligogalacturonides, damage-associated molecular patterns. Proc. Natl. Acad. Sci. U. S. A. 112, 5533–5538. 10.1073/pnas.1504154112, PMID: 25870275 PMC4418913

[ref20] Benedetti M. Verrascina I. Pontiggia D. Locci F. Mattei B. De Lorenzo G.. (2018). Four *Arabidopsis* berberine bridge enzyme-like proteins are specific oxidases that inactivate the elicitor-active oligogalacturonides. Plant J. 94, 260–273. 10.1111/tpj.13852, PMID: 29396998

[ref21] Beneloujaephajri E. Costa A. L’Haridon F. Métraux J. -P. Binda M. (2013). Production of reactive oxygen species and wound-induced resistance in *Arabidopsis thaliana* against *Botrytis cinerea* are preceded and depend on a burst of calcium. BMC Plant Biol. 13:160. 10.1186/1471-2229-13-160, PMID: 24134148 PMC4016300

[ref22] Bergey D. R. Orozco-Cardenas M. de Moura D. S. Ryan C. A. (1999). A wound- and systemin-inducible polygalacturonase in tomato leaves. Proc. Natl. Acad. Sci. U. S. A. 96, 1756–1760. 10.1073/pnas.96.4.1756, PMID: 9990097 PMC15585

[ref23] Bianchi M. E. Crippa M. P. Manfredi A. A. Mezzapelle R. Rovere Querini P. Venereau E. (2017). High-mobility group box 1 protein orchestrates responses to tissue damage *via* inflammation, innate and adaptive immunity, and tissue repair. Immunol. Rev. 280, 74–82. 10.1111/imr.12601, PMID: 29027228

[ref24] Bloch R. (1941). Wound healing in higher plants. Bot. Rev. 7, 110–146. 10.1007/BF02872446

[ref25] Blyth M. G. Morris R. J. (2019). Shear-enhanced dispersion of a wound substance as a candidate mechanism for variation potential transmission. Front. Plant Sci. 10:1393. 10.3389/fpls.2019.01393, PMID: 31803200 PMC6872641

[ref26] Boari F. Malone M. (1993). Wound-induced hydraulic signals: survey of occurrence in a range of species. J. Exp. Bot. 44, 741–746. 10.1093/jxb/44.4.741

[ref27] Bögre L. Ligterink W. Meskiene I. Barker P. J. Heberle-Bors E. Huskisson N. S.. (1997). Wounding induces the rapid and transient activation of a specific MAP kinase pathway. Plant Cell 9, 75–83. 10.1105/tpc.9.1.75, PMID: 12237344 PMC156902

[ref28] Boller T. Kende H. (1980). Regulation of wound ethylene synthesis in plants. Nature 286, 259–260. 10.1038/286259a0

[ref29] Bollhöner B. Zhang B. Stael S. Denancé N. Overmyer K. Goffner D.. (2013). *Post mortem* function of AtMC9 in xylem vessel elements. New Phytol. 200, 498–510. 10.1111/nph.12387, PMID: 23834670

[ref30] Bonaventure G. Gfeller A. Proebsting W. M. Hörtensteiner S. Chételat A. Martinoia E.. (2007). A gain-of-function allele of TPC1 activates oxylipin biogenesis after leaf wounding in *Arabidopsis*. Plant J. 49, 889–898. 10.1111/j.1365-313X.2006.03002.x, PMID: 17253984

[ref31] Bouwmeester H. Schuurink R. C. Bleeker P. M. Schiestl F. (2019). The role of volatiles in plant communication. Plant J. 100, 892–907. 10.1111/tpj.14496, PMID: 31410886 PMC6899487

[ref32] Bradley D. J. Kjellbom P. Lamb C. J. (1992). Elicitor- and wound-induced oxidative cross-linking of a proline-rich plant cell wall protein: a novel, rapid defense response. Cell 70, 21–30. 10.1016/0092-8674(92)90530-P, PMID: 1623521

[ref33] Broekaert W. F. Peumans W. J. (1988). Pectic polysaccharides elicit chitinase accumulation in tobacco. Physiol. Plant. 74, 740–744. 10.1111/j.1399-3054.1988.tb02046.x

[ref34] Browse J. (2009). Jasmonate passes muster: a receptor and targets for the defense hormone. Annu. Rev. Plant Biol. 60, 183–205. 10.1146/annurev.arplant.043008.092007, PMID: 19025383

[ref35] Brutus A. Sicilia F. Macone A. Cervone F. De Lorenzo G. (2010). A domain swap approach reveals a role of the plant wall-associated kinase 1 (WAK1) as a receptor of oligogalacturonides. Proc. Natl. Acad. Sci. U. S. A. 107, 9452–9457. 10.1073/pnas.1000675107, PMID: 20439716 PMC2889104

[ref36] Burdon-Sanderson J. S. (1873). Note on the electrical phenomena which accompany irritation of the leaf of *Dionæa muscipula*. Proc. R. Soc. Lond. 21, 495–496. 10.1098/rspl.1872.0092

[ref37] Cabrera J. C. Boland A. Messiaen J. Cambier P. Van Cutsem P. (2008). Egg box conformation of oligogalacturonides: the time-dependent stabilization of the elicitor-active conformation increases its biological activity. Glycobiology 18, 473–482. 10.1093/glycob/cwn027, PMID: 18381977

[ref38] Campos M. L. Yoshida Y. Major I. T. de Oliveira Ferreira D. Weraduwage S. M. Froehlich J. E.. (2016). Rewiring of jasmonate and phytochrome B signalling uncouples plant growth-defense tradeoffs. Nat. Commun. 7:12570. 10.1038/ncomms12570, PMID: 27573094 PMC5155487

[ref39] Canher B. Heyman J. Savina M. Devendran A. Eekhout T. Vercauteren I.. (2020). Rocks in the auxin stream: wound-induced auxin accumulation and *ERF115* expression synergistically drive stem cell regeneration. Proc. Natl. Acad. Sci. U. S. A. 117, 16667–16677. 10.1073/pnas.2006620117, PMID: 32601177 PMC7368246

[ref40] Cardoso A. A. McAdam S. A. M. (2019). Misleading conclusions from exogenous ABA application: a cautionary tale about the evolution of stomatal responses to changes in leaf water status. Plant Signal. Behav. 14:1610307. 10.1080/15592324.2019.1610307, PMID: 31032706 PMC6619974

[ref371] Chandra S. Low P. S. (1997). Measurement of Ca^2+^ fluxes during elicitation of the oxidative burst in aequorin-transformed tobacco cells. J. Biol. Chem. 272, 28274–28280. 10.1074/jbc.272.45.28274, PMID: 9353281

[ref41] Chassot C. Buchala A. Schoonbeek H. -J. Métraux J. -P. Lamotte O. (2008). Wounding of *Arabidopsis* leaves causes a powerful but transient protection against *Botrytis* infection. Plant J. 55, 555–567. 10.1111/j.1365-313X.2008.03540.x, PMID: 18452590

[ref42] Chauvin A. Caldelari D. Wolfender J. -L. Farmer E. E. (2013). Four 13-lipoxygenases contribute to rapid jasmonate synthesis in wounded *Arabidopsis thaliana* leaves: a role for lipoxygenase 6 in responses to long-distance wound signals. New Phytol. 197, 566–575. 10.1111/nph.12029, PMID: 23171345

[ref43] Chauvin A. Lenglet A. Wolfender J. -L. Farmer E. E. (2016). Paired hierarchical organization of 13-lipoxygenases in *Arabidopsis*. Plants 5:16. 10.3390/plants5020016, PMID: 27135236 PMC4931396

[ref44] Chen D. Cao Y. Li H. Kim D. Ahsan N. Thelen J.. (2017). Extracellular ATP elicits DORN1-mediated RBOHD phosphorylation to regulate stomatal aperture. Nat. Commun. 8:2265. 10.1038/s41467-017-02340-3, PMID: 29273780 PMC5741621

[ref45] Chen Y. -L. Fan K. -T. Hung S. -C. Chen Y. -R. (2020). The role of peptides cleaved from protein precursors in eliciting plant stress reactions. New Phytol. 225, 2267–2282. 10.1111/nph.16241, PMID: 31595506

[ref46] Chen Y. -L. Lee C. -Y. Cheng K. -T. Chang W. -H. Huang R. -N. Nam H. G.. (2014). Quantitative peptidomics study reveals that a wound-induced peptide from PR-1 regulates immune signaling in tomato. Plant Cell 26, 4135–4148. 10.1105/tpc.114.131185, PMID: 25361956 PMC4247587

[ref48] Chen Y. -C. Siems W. F. Pearce G. Ryan C. A. (2008). Six peptide wound signals derived from a single precursor protein in *Ipomoea batatas* leaves activate the expression of the defense gene sporamin. J. Biol. Chem. 283, 11469–11476. 10.1074/jbc.M709002200, PMID: 18299332 PMC2431084

[ref49] Cheval C. Faulkner C. (2018). Plasmodesmal regulation during plant-pathogen interactions. New Phytol. 217, 62–67. 10.1111/nph.14857, PMID: 29083038

[ref50] Chichkova N. V. Shaw J. Galiullina R. A. Drury G. E. Tuzhikov A. I. Kim S. H.. (2010). Phytaspase, a relocalisable cell death promoting plant protease with caspase specificity. EMBO J. 29, 1149–1161. 10.1038/emboj.2010.1, PMID: 20111004 PMC2845272

[ref51] Chien P. -S. Nam H. G. Chen Y. -R. (2015). A salt-regulated peptide derived from the CAP superfamily protein negatively regulates salt-stress tolerance in *Arabidopsis*. J. Exp. Bot. 66, 5301–5313. 10.1093/jxb/erv263, PMID: 26093145 PMC4526916

[ref52] Chini A. Fonseca S. Fernández G. Adie B. Chico J. M. Lorenzo O.. (2007). The JAZ family of repressors is the missing link in jasmonate signalling. Nature 448, 666–671. 10.1038/nature06006, PMID: 17637675

[ref53] Chini A. Gimenez-Ibanez S. Goossens A. Solano R. (2016). Redundancy and specificity in jasmonate signalling. Curr. Opin. Plant Biol. 33, 147–156. 10.1016/j.pbi.2016.07.005, PMID: 27490895

[ref54] Chini A. Monte I. Zamarreño A. M. Hamberg M. Lassueur S. Reymond P.. (2018). An OPR3-independent pathway uses 4,5-didehydrojasmonate for jasmonate synthesis. Nat. Chem. Biol. 14, 171–178. 10.1038/nchembio.2540, PMID: 29291349

[ref55] Chivasa S. Murphy A. M. Hamilton J. M. Lindsey K. Carr J. P. Slabas A. R. (2009). Extracellular ATP is a regulator of pathogen defence in plants. Plant J. 60, 436–448. 10.1111/j.1365-313X.2009.03968.x, PMID: 19594709

[ref56] Chivasa S. Ndimba B. K. Simon W. J. Lindsey K. Slabas A. R. (2005). Extracellular ATP functions as an endogenous external metabolite regulating plant cell viability. Plant Cell 17, 3019–3034. 10.1105/tpc.105.036806, PMID: 16199612 PMC1276027

[ref57] Choi H. W. Klessig D. F. (2016). DAMPs, MAMPs, and NAMPs in plant innate immunity. BMC Plant Biol. 16:232. 10.1186/s12870-016-0921-2, PMID: 27782807 PMC5080799

[ref58] Choi H. W. Manohar M. Manosalva P. Tian M. Moreau M. Klessig D. F. (2016). Activation of plant innate immunity by extracellular High Mobility Group Box 3 and its inhibition by salicylic acid. PLoS Pathog. 12:e1005518. 10.1371/journal.ppat.1005518, PMID: 27007252 PMC4805298

[ref59] Choi W.-G. Miller G. Wallace I. Harper J. Mittler R. Gilroy S. (2017). Orchestrating rapid long-distance signaling in plants with Ca^2+^, ROS and electrical signals. Plant J. 90, 698–707. 10.1111/tpj.13492, PMID: 28112437 PMC5677518

[ref60] Choi J. Tanaka K. Cao Y. Qi Y. Qiu J. Liang Y.. (2014). Identification of a plant receptor for extracellular ATP. Science 343, 290–294. 10.1126/science.343.6168.290, PMID: 24436418

[ref61] Choi W.-G. Toyota M. Kim S. H. Hilleary R. Gilroy S. (2014). Salt stress-induced Ca^2+^ waves are associated with rapid, long-distance root-to-shoot signaling in plants. Proc. Natl. Acad. Sci. U. S. A. 111, 6497–6502. 10.1073/pnas.1319955111, PMID: 24706854 PMC4035928

[ref62] Cisneros-Zevallos L. Jacobo-Velázquez D. A. Pech J. -C. Koiwa H. (2014). “Signaling molecules involved in the postharvest stress response of plants quality changes and synthesis of secondary metabolites” in Handbook of plant and crop physiology. 3rd Edn. ed. Pessarakli M. (Boca Raton: CRC Press), 259–276.

[ref63] Clark G. Torres J. Finlayson S. Guan X. Handley C. Lee J.. (2010). Apyrase (nucleoside triphosphate-diphosphohydrolase) and extracellular nucleotides regulate cotton fiber elongation in cultured ovules. Plant Physiol. 152, 1073–1083. 10.1104/pp.109.147637, PMID: 20018604 PMC2815863

[ref64] Claverie J. Balacey S. Lemaître-Guillier C. Brulé D. Chiltz A. Granet L.. (2018). The cell wall-derived xyloglucan is a new DAMP triggering plant immunity in *Vitis vinifera* and *Arabidopsis thaliana*. Front. Plant Sci. 9:1725. 10.3389/fpls.2018.01725, PMID: 30546374 PMC6280107

[ref65] Consales F. Schweizer F. Erb M. Gouhier-Darimont C. Bodenhausen N. Bruessow F.. (2012). Insect oral secretions suppress wound-induced responses in *Arabidopsis*. J. Exp. Bot. 63, 727–737. 10.1093/jxb/err308, PMID: 21994175 PMC3254683

[ref66] Coolen S. Van Pelt J. A. Van Wees S. C. M. Pieterse C. M. J. (2019). Mining the natural genetic variation in *Arabidopsis thaliana* for adaptation to sequential abiotic and biotic stresses. Planta 249, 1087–1105. 10.1007/s00425-018-3065-9, PMID: 30547240

[ref67] Coppola M. Di Lelio I. Romanelli A. Gualtieri L. Molisso D. Ruocco M.. (2019). Tomato plants treated with systemin peptide show enhanced levels of direct and indirect defense associated with increased expression of defense-related genes. Plants 8:395. 10.3390/plants8100395, PMID: 31623335 PMC6843623

[ref68] Corpas F. J. Chaki M. Fernández-Ocaña A. Valderrama R. Palma J. M. Carreras A.. (2008). Metabolism of reactive nitrogen species in pea plants under abiotic stress conditions. Plant Cell Physiol. 49, 1711–1722. 10.1093/pcp/pcn144, PMID: 18801763

[ref69] Costa A. Luoni L. Marrano C. A. Hashimoto K. Köster P. Giacometti S.. (2017). Ca^2+^-dependent phosphoregulation of the plasma membrane Ca^2+^-ATPase ACA8 modulates stimulus-induced calcium signatures. J. Exp. Bot. 68, 3215–3230. 10.1093/jxb/erx162, PMID: 28531251 PMC5853299

[ref70] Cui F. Brosché M. Lehtonen M. T. Amiryousefi A. Xu E. Punkkinen M.. (2016). Dissecting abscisic acid signaling pathways involved in cuticle formation. Mol. Plant 9, 926–938. 10.1016/j.molp.2016.04.001, PMID: 27060495

[ref71] Cui F. Brosché M. Sipari N. Tang S. Overmyer K. (2013). Regulation of ABA dependent wound induced spreading cell death by MYB108. New Phytol. 200, 634–640. 10.1111/nph.12456, PMID: 23952703

[ref72] Cui W. Lee J. Y. (2016). *Arabidopsis* callose synthases CalS1/8 regulate plasmodesmal permeability during stress. Nat. Plants 2:16034. 10.1038/NPLANTS.2016.34, PMID: 27243643

[ref73] Cui F. Wu W. Wang K. Zhang Y. Hu Z. Brosché M.. (2019). Cell death regulation but not abscisic acid signaling is required for enhanced immunity to *Botrytis* in *Arabidopsis* cuticle-permeable mutants. J. Exp. Bot. 70, 5971–5984. 10.1093/jxb/erz345, PMID: 31328223 PMC6812726

[ref74] Cuin T. A. Dreyer I. Michard E. (2018). The role of potassium channels in *Arabidopsis thaliana* long distance electrical signalling: AKT2 modulates tissue excitability while GORK shapes action potentials. Int. J. Mol. Sci. 19:926. 10.3390/ijms19040926, PMID: 29561764 PMC5979599

[ref75] Dammann C. Rojo E. Sánchez-Serrano J. J. (1997). Abscisic acid and jasmonic acid activate wound-inducible genes in potato through separate, organ-specific signal transduction pathways. Plant J. 11, 773–782. 10.1046/j.1365-313x.1997.11040773.x, PMID: 9161035

[ref76] Daniel B. Konrad B. Toplak M. Lahham M. Messenlehner J. Winkler A.. (2017). The family of berberine bridge enzyme-like enzymes: a treasure-trove of oxidative reactions. Arch. Biochem. Biophys. 632, 88–103. 10.1016/j.abb.2017.06.023, PMID: 28676375

[ref77] Davière J. -M. Achard P. (2016). A pivotal role of DELLAs in regulating multiple hormone signals. Mol. Plant 9, 10–20. 10.1016/j.molp.2015.09.011, PMID: 26415696

[ref78] Davies E. (2006). “Electrical signals in plants: facts and hypotheses” in Plant electrophysiology: Theory and methods. ed. Volkov A. G. (Berlin: Springer), 407–422.

[ref79] Davis K. R. Hahlbrock K. (1987). Induction of defense responses in cultured parsley cells by plant cell wall fragments. Plant Physiol. 84, 1286–1290. 10.1104/pp.84.4.1286, PMID: 16665599 PMC1056766

[ref80] de Azevedo Souza C. Li S. Lin A. Z. Boutrot F. Grossmann G. Zipfel C.. (2017). Cellulose-derived oligomers act as damage-associated molecular patterns and trigger defense-like responses. Plant Physiol. 173, 2383–2398. 10.1104/pp.16.01680, PMID: 28242654 PMC5373054

[ref81] De Boer K. Tilleman S. Pauwels L. Vanden Bossche R. De Sutter V. Vanderhaeghen R.. (2011). APETALA2/ETHYLENE RESPONSE FACTOR and basic helix-loop-helix tobacco transcription factors cooperatively mediate jasmonate-elicited nicotine biosynthesis. Plant J. 66, 1053–1065. 10.1111/j.1365-313X.2011.04566.x, PMID: 21418355

[ref82] De Lorenzo G. Ferrari S. Cervone F. Okun E. (2018). Extracellular DAMPs in plants and mammals: immunity, tissue damage and repair. Trends Immunol. 39, 937–950. 10.1016/j.it.2018.09.006, PMID: 30293747

[ref83] De Vriese K. Costa A. Beeckman T. Vanneste S. (2018). Pharmacological strategies for manipulating plant Ca^2+^ signalling. Int. J. Mol. Sci. 19:1506. 10.3390/ijms19051506, PMID: 29783646 PMC5983822

[ref84] Decreux A. Messiaen J. (2005). Wall-associated kinase WAK1 interacts with cell wall pectins in a calcium-induced conformation. Plant Cell Physiol. 46, 268–278. 10.1093/pcp/pci026, PMID: 15769808

[ref85] Delessert C. Wilson I. W. Van Der Straeten D. Dennis E. S. Dolferus R. (2004). Spatial and temporal analysis of the local response to wounding in *Arabidopsis* leaves. Plant Mol. Biol. 55, 165–181. 10.1007/s11103-004-0112-7, PMID: 15604673

[ref372] Demidchik V. Shang Z. Shin R. Thompson E. Rubio L. Laohavisit A.. (2009). Plant extracellular ATP signalling by plasma membrane NADPH oxidase and Ca^2+^ channels. Plant J. 58, 903–913. 10.1111/j.1365-313X.2009.03830.x, PMID: 19220789

[ref86] Deng S. Sun J. Zhao R. Ding M. Zhang Y. Sun Y.. (2015). *Populus euphratica* APYRASE2 enhances cold tolerance by modulating vesicular trafficking and extracellular ATP in *Arabidopsis* plants. Plant Physiol. 169, 530–548. 10.1104/pp.15.00581, PMID: 26224801 PMC4577398

[ref87] Denoux C. Galletti R. Mammarella N. Gopalan S. Werck D. De Lorenzo G.. (2008). Activation of defense response pathways by OGs and Flg22 elicitors in *Arabidopsis* seedlings. Mol. Plant 1, 423–445. 10.1093/mp/ssn019, PMID: 19825551 PMC2954645

[ref88] Deutsch C. A. Tewksbury J. J. Tigchelaar M. Battisti D. S. Merrill S. C. Huey R. B.. (2018). Increase in crop losses to insect pests in a warming climate. Science 361, 916–919. 10.1126/science.aat3466, PMID: 30166490

[ref89] Dobney S. Chiasson D. Lam P. Smith S. P. Snedden W. A. (2009). The calmodulin-related calcium sensor CML42 plays a role in trichome branching. J. Biol. Chem. 284, 31647–31657. 10.1074/jbc.M109.056770, PMID: 19720824 PMC2797235

[ref90] Dodd A. N. Kudla J. Sanders D. (2010). The language of calcium signaling. Annu. Rev. Plant Biol. 61, 593–620. 10.1146/annurev-arplant-070109-104628, PMID: 20192754

[ref91] Doke N. Miura Y. Chai H. -B. Kawakita K. (1991). “Involvement of active oxygen in induction of plant defense response against infection and injury” in Active oxygen/oxidative stress and plant metabolism. eds. Pell E. Steffen K. (Rockville: American Society of Plant Physiologists), 84–96.

[ref92] D’Ovidio R. Mattei B. Roberti S. Bellincampi D. (2004). Polygalacturonases, polygalacturonase-inhibiting proteins and pectic oligomers in plant-pathogen interactions. Biochim. Biophys. Acta 1696, 237–244. 10.1016/j.bbapap.2003.08.012, PMID: 14871664

[ref93] Dubiella U. Seybold H. Durian G. Komander E. Lassig R. Witte C. -P.. (2013). Calcium-dependent protein kinase/NADPH oxidase activation circuit is required for rapid defense signal propagation. Proc. Natl. Acad. Sci. U. S. A. 110, 8744–8749. 10.1073/pnas.1221294110, PMID: 23650383 PMC3666735

[ref95] Duran-Flores D. Heil M. (2016). Sources of specificity in plant damaged-self recognition. Curr. Opin. Plant Biol. 32, 77–87. 10.1016/j.pbi.2016.06.019, PMID: 27421107

[ref96] Duran-Flores D. Heil M. (2018). Extracellular self-DNA as a damage-associated molecular pattern (DAMP) that triggers self-specific immunity induction in plants. Brain Behav. Immun. 72, 78–88. 10.1016/j.bbi.2017.10.010, PMID: 29042243

[ref97] Dziubinska H. Filek M. Koscielniak J. Trebacz K. (2003). Variation and action potentials evoked by thermal stimuli accompany enhancement of ethylene emission in distant non-stimulated leaves of *Vicia faba minor* seedlings. J. Plant Physiol. 160, 1203–1210. 10.1078/0176-1617-00914, PMID: 14610889

[ref98] Efroni I. Mello A. Nawy T. Ip P. -L. Rahni R. DelRose N.. (2016). Root regeneration triggers an embryo-like sequence guided by hormonal interactions. Cell 165, 1721–1733. 10.1016/j.cell.2016.04.046, PMID: 27212234 PMC4912400

[ref99] Engelsdorf T. Gigli-Bisceglia N. Veerabagu M. McKenna J. F. Vaahtera L. Augstein F.. (2018). The plant cell wall integrity maintenance and immune signaling systems cooperate to control stress responses in *Arabidopsis thaliana*. Sci. Signal. 11:eaao3070. 10.1126/scisignal.aao3070, PMID: 29945884

[ref100] Erb M. Reymond P. (2019). Molecular interactions between plants and insect herbivores. Annu. Rev. Plant Biol. 70, 527–557. 10.1146/annurev-arplant-050718-095910, PMID: 30786233

[ref101] Escamez S. Stael S. Vainonen J. P. Willems P. Jin H. Kimura S.. (2019). Extracellular peptide Kratos restricts cell death during vascular development and stress in *Arabidopsis*. J. Exp. Bot. 70, 2199–2210. 10.1093/jxb/erz021, PMID: 30753577 PMC6460963

[ref102] Evans M. J. Choi W. -G. Gilroy S. Morris R. J. (2016). A ROS-assisted calcium wave dependent on the AtRBOHD NADPH oxidase and TPC1 cation channel propagates the systemic response to salt stress. Plant Physiol. 171, 1771–1784. 10.1104/pp.16.00215, PMID: 27261066 PMC4936552

[ref103] Evans M. J. Morris R. J. (2017). Chemical agents transported by xylem mass flow propagate variation potentials. Plant J. 91, 1029–1037. 10.1111/tpj.13624, PMID: 28656705 PMC5601289

[ref104] Farmer E. E. Gao Y. -Q. Lenzoni G. Wolfender J. -L. Wu Q. (2020). Wound- and mechanostimulated electrical signals control hormone responses. New Phytol. 227, 1037–1050. 10.1111/nph.16646, PMID: 32392391

[ref105] Favre P. Agosti R. D. (2007). Voltage-dependent action potentials in *Arabidopsis thaliana*. Physiol. Plant. 131, 263–272. 10.1111/j.1399-3054.2007.00954.x, PMID: 18251897

[ref106] Fernández-Milmanda G. L. Crocco C. D. Reichelt M. Mazza C. A. Köllner T. G. Zhang T.. (2020). A light-dependent molecular link between competition cues and defence responses in plants. Nat. Plants 6, 223–230. 10.1038/s41477-020-0604-8, PMID: 32170284

[ref107] Ferrari S. Savatin D. V. Sicilia F. Gramegna G. Cervone F. De Lorenzo G. (2013). Oligogalacturonides: plant damage-associated molecular patterns and regulators of growth and development. Front. Plant Sci. 4:49. 10.3389/fpls.2013.00049, PMID: 23493833 PMC3595604

[ref108] Fichman Y. Miller G. Mittler R. (2019). Whole-plant live imaging of reactive oxygen species. Mol. Plant 12, 1203–1210. 10.1016/j.molp.2019.06.003, PMID: 31220601

[ref109] Fleurat-Lessard P. Bouché-Pillon S. Leloup C. Bonnemain J. -L. (1997). Distribution and activity of the plasma membrane H^+^-ATPase in *Mimosa pudica* L. in relation to ionic fluxes and leaf movements. Plant Physiol. 113, 747–754. 10.1104/pp.113.3.747, PMID: 12223640 PMC158192

[ref373] Flury P. Klauser D. Schulze B. Boller T. Bartels S. (2013). The anticipation of danger: microbe-associated molecular pattern perception enhances AtPep-triggered oxidative burst. Plant Physiol. 161, 2023–2035. 10.1104/pp.113.216077, PMID: 23400703 PMC3613473

[ref110] Fonseca S. Chico J. M. Solano R. (2009). The jasmonate pathway: the ligand, the receptor and the core signalling module. Curr. Opin. Plant Biol. 12, 539–547. 10.1016/j.pbi.2009.07.013, PMID: 19716757

[ref111] Förster S. Schmidt L. K. Kopic E. Anschütz U. Huang S. Schlücking K.. (2019). Wounding-induced stomatal closure requires jasmonate-mediated activation of GORK K^+^ channels by a Ca^2+^ sensor-kinase CBL1-CIPK5 complex. Dev. Cell 48, 87–99. 10.1016/j.devcel.2018.11.014, PMID: 30528785

[ref112] Fürstenberg-Hägg J. Zagrobelny M. Bak S. (2013). Plant defense against insect herbivores. Int. J. Mol. Sci. 14, 10242–10297. 10.3390/ijms140510242, PMID: 23681010 PMC3676838

[ref113] Galletti R. Denoux C. Gambetta S. Dewdney J. Ausubel F. M. De Lorenzo G.. (2008). The AtrbohD-mediated oxidative burst elicited by oligogalacturonides in *Arabidopsis* is dispensable for the activation of defense responses effective against *Botrytis cinerea*. Plant Physiol. 148, 1695–1706. 10.1104/pp.108.127845, PMID: 18790995 PMC2577270

[ref114] García T. Gutiérrez J. Veloso J. Gago-Fuentes R. Díaz J. (2015). Wounding induces local resistance but systemic susceptibility to *Botrytis cinerea* in pepper plants. J. Plant Physiol. 176, 202–209. 10.1016/j.jplph.2014.12.013, PMID: 25662842

[ref115] Gasperini D. Chételat A. Acosta I. F. Goossens J. Pauwels L. Goossens A.. (2015). Multilayered organization of jasmonate signalling in the regulation of root growth. PLoS Genet. 11:e1005300. 10.1371/journal.pgen.1005300, PMID: 26070206 PMC4466561

[ref116] Gilroy S. Białasek M. Suzuki N. Górecka M. Devireddy A. R. Karpiński S.. (2016). ROS, calcium, and electric signals: key mediators of rapid systemic signaling in plants. Plant Physiol. 171, 1606–1615. 10.1104/pp.16.00434, PMID: 27208294 PMC4936577

[ref117] Glauser G. Grata E. Dubugnon L. Rudaz S. Farmer E. E. Wolfender J. -L. (2008). Spatial and temporal dynamics of jasmonate synthesis and accumulation in *Arabidopsis* in response to wounding. J. Biol. Chem. 283, 16400–16407. 10.1074/jbc.M801760200, PMID: 18400744

[ref118] Gong T. Liu L. Jiang W. Zhou R. (2020). DAMP-sensing receptors in sterile inflammation and inflammatory diseases. Nat. Rev. Immunol. 20, 95–112. 10.1038/s41577-019-0215-7, PMID: 31558839

[ref119] Goossens J. Fernández-Calvo P. Schweizer F. Goossens A. (2016). Jasmonates: signal transduction components and their roles in environmental stress responses. Plant Mol. Biol. 91, 673–689. 10.1007/s11103-016-0480-9, PMID: 27086135

[ref374] Goto Y. Maki N. Ichihashi Y. Kitazawa D. Igarashi D. Kadota Y.. (2020). Exogenous treatment with glutamate induces immune responses in *Arabidopsis*. Mol. Plant-Microbe Interact. 33, 474–487. 10.1094/MPMI-09-19-0262-R, PMID: 31721650

[ref120] Gramegna G. Modesti V. Savatin D. V. Sicilia F. Cervone F. De Lorenzo G. (2016). *GRP-3* and *KAPP*, encoding interactors of WAK1, negatively affect defense responses induced by oligogalacturonides and local response to wounding. J. Exp. Bot. 67, 1716–1729. 10.1093/jxb/erv563, PMID: 26748394 PMC4783359

[ref121] Grebner W. Stingl N. E. Oenel A. Mueller M. J. Berger S. (2013). Lipoxygenase6-dependent oxylipin synthesis in roots is required for abiotic and biotic stress resistance of *Arabidopsis*. Plant Physiol. 161, 2159–2170. 10.1104/pp.113.214544, PMID: 23444343 PMC3613484

[ref122] Guan L. Denkert N. Eisa A. Lehmann M. Sjuts I. Weiberg A.. (2019). JASSY, a chloroplast outer membrane protein required for jasmonate biosynthesis. Proc. Natl. Acad. Sci. U. S. A. 116, 10568–10575. 10.1073/pnas.1900482116, PMID: 31068459 PMC6534988

[ref123] Guo Q. Yoshida Y. Major I. T. Wang K. Sugimoto K. Kapali G.. (2018). JAZ repressors of metabolic defense promote growth and reproductive fitness in *Arabidopsis*. Proc. Natl. Acad. Sci. U. S. A. 115, E10768–E10777. 10.1073/pnas.1811828115, PMID: 30348775 PMC6233084

[ref124] Gust A. A. Pruitt R. Nürnberger T. (2017). Sensing danger: key to activating plant immunity. Trends Plant Sci. 22, 779–791. 10.1016/j.tplants.2017.07.005, PMID: 28779900

[ref125] Haag F. Adriouch S. Braß A. Jung C. Möller S. Scheuplein F.. (2007). Extracellular NAD and ATP: partners in immune cell modulation. Purinergic Signal 3, 71–81. 10.1007/s11302-006-9038-7, PMID: 18404420 PMC2096762

[ref126] Hadiarto T. Nanmori T. Matsuoka D. Iwasaki T. Sato K. I. Fukami Y.. (2006). Activation of *Arabidopsis* MAPK kinase kinase (AtMEKK1) and induction of AtMEKK1-AtMEK1 pathway by wounding. Planta 223, 708–713. 10.1007/s00425-005-0126-7, PMID: 16211390

[ref127] Hadwiger L. A. Tanaka K. (2018). DNA damage and chromatin conformation changes confer nonhost resistance: a hypothesis based on effects of anti-cancer agents on plant defense responses. Front. Plant Sci. 9:1056. 10.3389/fpls.2018.01056, PMID: 30087685 PMC6066612

[ref128] Hander T. Fernández-Fernández Á. D. Kumpf R. P. Willems P. Schatowitz H. Rombaut D.. (2019). Damage on plants activates Ca^2+^-dependent metacaspases for release of immunomodulatory peptides. Science 363:eaar7486. 10.1126/science.aar7486, PMID: 30898901

[ref129] He Y. Hong G. Zhang H. Tan X. Li L. Kong Y.. (2020). The OsGSK2 kinase integrates brassinosteroid and jasmonic acid signaling by interacting with OsJAZ4. Plant Cell 32, 2806–2822. 10.1105/tpc.19.00499, PMID: 32586913 PMC7474301

[ref130] Heil M. Ibarra-Laclette E. Adame-Álvarez R. M. Martínez O. Ramirez-Chávez E. Molina-Torres J.. (2012). How plants sense wounds: damaged-self recognition is based on plant-derived elicitors and induces octadecanoid signaling. PLoS One 7:e30537. 10.1371/journal.pone.0030537, PMID: 22347382 PMC3276496

[ref131] Heil M. Land W. G. (2014). Danger signals—damaged-self recognition across the tree of life. Front. Plant Sci. 5:578. 10.3389/fpls.2014.00578, PMID: 25400647 PMC4215617

[ref132] Heil M. Vega-Muñoz I. (2019). Nucleic acid sensing in mammals and plants: facts and caveats. Int. Rev. Cell Mol. Biol. 345, 225–285. 10.1016/bs.ircmb.2018.10.003, PMID: 30904194

[ref133] Heyman J. Canher B. Bisht A. Christiaens F. De Veylder L. (2018). Emerging role of the plant ERF transcription factors in coordinating wound defense responses and repair. J. Cell Sci. 131:jcs208215. 10.1242/jcs.208215, PMID: 29242229

[ref134] Heyman J. Cools T. Canher B. Shavialenka S. Traas J. Vercauteren I.. (2016). The heterodimeric transcription factor complex ERF115-PAT1 grants regeneration competence. Nat. Plants 2:16165. 10.1038/nplants.2016.165, PMID: 27797356

[ref135] Heyman J. Cools T. Vandenbussche F. Heyndrickx K. S. Van Leene J. Vercauteren I.. (2013). ERF115 controls root quiescent center cell division and stem cell replenishment. Science 342, 860–863. 10.1126/science.1240667, PMID: 24158907

[ref136] Hickman R. Van Verk M. C. Van Dijken A. J. H. Pereira Mendes M. Vroegop-Vos I. A. Caarls L.. (2017). Architecture and dynamics of the jasmonic acid gene regulatory network. Plant Cell 29, 2086–2105. 10.1105/tpc.16.00958, PMID: 28827376 PMC5635973

[ref137] Hoermayer L. Friml J. (2019). Targeted cell ablation-based insights into wound healing and restorative patterning. Curr. Opin. Plant Biol. 52, 124–130. 10.1016/j.pbi.2019.08.006, PMID: 31585333 PMC6900583

[ref138] Hoermayer L. Montesinos J. C. Marhava P. Benková E. Yoshida S. Friml J. (2020). Wounding-induced changes in cellular pressure and localized auxin signalling spatially coordinate restorative divisions in roots. Proc. Natl. Acad. Sci. U. S. A. 117, 15322–15331. 10.1073/pnas.2003346117, PMID: 32541049 PMC7334516

[ref139] Holdaway-Clarke T. L. Walker N. A. Hepler P. K. Overall R. L. (2000). Physiological elevations in cytoplasmic free calcium by cold or ion injection result in transient closure of higher plant plasmodesmata. Planta 210, 329–335. 10.1007/PL00008141, PMID: 10664140

[ref140] Hong J. H. Savina M. Du J. Devendran A. Kannivadi Ramakanth K. Tian X.. (2017). A sacrifice-for-survival mechanism protects root stem cell niche from chilling stress. Cell 170, 102–113. 10.1016/j.cell.2017.06.002, PMID: 28648662

[ref141] Horbach R. Navarro-Quesada A. R. Knogge W. Deising H. B. (2011). When and how to kill a plant cell: infection strategies of plant pathogenic fungi. J. Plant Physiol. 168, 51–62. 10.1016/j.jplph.2010.06.014, PMID: 20674079

[ref142] Hou X. Lee L. Y. C. Xia K. Yan Y. Yu H. (2010). DELLAs modulate jasmonate signaling *via* competitive binding to JAZs. Dev. Cell 19, 884–894. 10.1016/j.devcel.2010.10.024, PMID: 21145503

[ref143] Hou S. Liu Z. Shen H. Wu D. (2019). Damage-associated molecular pattern-triggered immunity in plants. Front. Plant Sci. 10:646. 10.3389/fpls.2019.00646, PMID: 31191574 PMC6547358

[ref375] Howe G. A. Lee G. I. Itoh A. Li L. DeRocher A. E. (2000). Cytochrome P450-dependent metabolism of oxylipins in tomato. Cloning and expression of allene oxide synthase and fatty acid hydroperoxide lyase. Plant Physiol. 123, 711–724. 10.1104/pp.123.2.711, PMID: 10859201 PMC59039

[ref144] Howe G. A. Major I. T. Koo A. J. (2018). Modularity in jasmonate signaling for multistress resilience. Annu. Rev. Plant Biol. 69, 387–415. 10.1146/annurev-arplant-042817-040047, PMID: 29539269

[ref145] Hu Z. Cools T. De Veylder L. (2016). Mechanisms used by plants to cope with DNA damage. Annu. Rev. Plant Biol. 67, 439–462. 10.1146/annurev-arplant-043015-111902, PMID: 26653616

[ref146] Hu X. Xu L. (2016). Transcription factors WOX11/12 directly activate *WOX5/7* to promote root primordia initiation and organogenesis. Plant Physiol. 172, 2363–2373. 10.1104/pp.16.01067, PMID: 27784768 PMC5129711

[ref147] Huang H. -J. Cui J. -R. Xia X. Chen J. Ye Y. -X. Zhang C. -X.. (2019). Salivary DNase II from *Laodelphax striatellus* acts as an effector that suppresses plant defence. New Phytol. 224, 860–874. 10.1111/nph.15792, PMID: 30883796

[ref148] Huang X. Stettmaier K. Michel C. Hutzler P. Mueller M. J. Durner J. (2004). Nitric oxide is induced by wounding and influences jasmonic acid signaling in *Arabidopsis thaliana*. Planta 218, 938–946. 10.1007/s00425-003-1178-1, PMID: 14716563

[ref149] Huffaker A. Pearce G. Ryan C. A. (2006). An endogenous peptide signal in *Arabidopsis* activates components of the innate immune response. Proc. Natl. Acad. Sci. U. S. A. 103, 10098–10103. 10.1073/pnas.0603727103, PMID: 16785434 PMC1502512

[ref150] Huffaker A. Pearce G. Veyrat N. Erb M. Turlings T. C. J. Sartor R.. (2013). Plant elicitor peptides are conserved signals regulating direct and indirect antiherbivore defense. Proc. Natl. Acad. Sci. U. S. A. 110, 5707–5712. 10.1073/pnas.1214668110, PMID: 23509266 PMC3619339

[ref376] Huffaker A. Ryan C. A. (2007). Endogenous peptide defense signals in *Arabidopsis* differentially amplify signaling for the innate immune response. Proc. Natl. Acad. Sci. U. S. A. 104, 10732–10736. 10.1073/pnas.0703343104, PMID: 17566109 PMC1965581

[ref151] Huot B. Yao J. Montgomery B. L. He S. Y. (2014). Growth-defense tradeoffs in plants: a balancing act to optimize fitness. Mol. Plant 7, 1267–1287. 10.1093/mp/ssu049, PMID: 24777989 PMC4168297

[ref152] Hussein Z. Fawole O. A. Opara U. L. (2020). Harvest and postharvest factors affecting bruise damage of fresh fruits. Hortic. Plant J. 6, 1–13. 10.1016/j.hpj.2019.07.006

[ref153] Iakimova E. T. Woltering E. J. (2018). The wound response in fresh-cut lettuce involves programmed cell death events. Protoplasma 255, 1225–1238. 10.1007/s00709-018-1228-y, PMID: 29470708 PMC5994216

[ref154] Ichimura K. Mizoguchi T. Yoshida R. Yuasa T. Shinozaki K. (2000). Various abiotic stresses rapidly activate *Arabidopsis* MAP kinases ATMPK4 and ATMPK6. Plant J. 24, 655–665. 10.1046/j.1365-313X.2000.00913.x, PMID: 11123804

[ref155] Ikeuchi M. Iwase A. Rymen B. Lambolez A. Kojima M. Takebayashi Y.. (2017). Wounding triggers callus formation *via* dynamic hormonal and transcriptional changes. Plant Physiol. 175, 1158–1174. 10.1104/pp.17.01035, PMID: 28904073 PMC5664475

[ref156] Ikeuchi M. Sugimoto K. Iwase A. (2013). Plant callus: mechanisms of induction and repression. Plant Cell 25, 3159–3173. 10.1105/tpc.113.116053/, PMID: 24076977 PMC3809525

[ref157] Iwase A. Mitsuda N. Koyama T. Hiratsu K. Kojima M. Arai T.. (2011). The AP2/ERF transcription factor WIND1 controls cell dedifferentiation in *Arabidopsis*. Curr. Biol. 21, 508–514. 10.1016/j.cub.2011.02.020, PMID: 21396822

[ref158] Jacobs A. K. Lipka V. Burton R. A. Panstruga R. Strizhov N. Schulze-Lefert P.. (2003). An *Arabidopsis* callose synthase, GSL5, is required for wound and papillary callose formation. Plant Cell 15, 2503–2513. 10.1105/tpc.016097, PMID: 14555698 PMC280557

[ref159] Jewell J. B. Tanaka K. (2019). Transcriptomic perspective on extracellular ATP signaling: a few curious trifles. Plant Signal. Behav. 14:e1659079. 10.1080/15592324.2019.1659079, PMID: 31451022 PMC6804718

[ref160] Jing Y. Shen N. Zheng X. Fu A. Zhao F. Lan W.. (2020). Danger-associated peptide regulates root immune responses and root growth by affecting ROS formation in *Arabidopsis*. Int. J. Mol. Sci. 21:4590. 10.3390/ijms21134590, PMID: 32605179 PMC7369728

[ref161] Johnson R. A. Conklin P. A. Tjahjadi M. Missirian V. Toal T. Brady S. M.. (2018). SUPPRESSOR OF GAMMA RESPONSE1 links DNA damage response to organ regeneration. Plant Physiol. 176, 1665–1675. 10.1104/pp.17.01274, PMID: 29222192 PMC5813563

[ref162] Kadota Y. Sklenar J. Derbyshire P. Stransfeld L. Asai S. Ntoukakis V.. (2014). Direct regulation of the NADPH oxidase RBOHD by the PRR-associated kinase BIK1 during plant immunity. Mol. Cell 54, 43–55. 10.1016/j.molcel.2014.02.021, PMID: 24630626

[ref163] Kanchiswamy C. N. Takahashi H. Quadro S. Maffei M. E. Bossi S. Bertea C.. (2010). Regulation of *Arabidopsis* defense responses against *Spodoptera littoralis* by CPK-mediated calcium signaling. BMC Plant Biol. 10:97. 10.1186/1471-2229-10-97, PMID: 20504319 PMC3095362

[ref164] Kato M. Hayakawa Y. Hyodo H. Ikoma Y. Yano M. (2000). Wound-induced ethylene synthesis and expression and formation of 1-aminocyclopropane-1-carboxylate (ACC) synthase, ACC oxidase, phenylalanine ammonia-lyase, and peroxidase in wounded mesocarp tissue of *Cucurbita maxima*. Plant Cell Physiol. 41, 440–447. 10.1093/pcp/41.4.440, PMID: 10845457

[ref165] Kauss H. Köhle H. Jeblick W. (1983). Proteolytic activation and stimulation by Ca^2+^ of glucan synthase from soybean cells. FEBS Lett. 158, 84–88. 10.1016/0014-5793(83)80681-4

[ref166] Kenton P. Mur L. A. J. Draper J. (1999). A requirement for calcium and protein phosphatase in the jasmonate-induced increase in tobacco leaf acid phosphatase specific activity. J. Exp. Bot. 50, 1331–1341. 10.1093/jxb/50.337.1331

[ref167] Kessler A. Baldwin I. T. (2001). Defensive function of herbivore-induced plant volatile emissions in nature. Science 291, 2141–2144. 10.1126/science.291.5511.2141, PMID: 11251117

[ref168] Kiep V. Vadassery J. Lattke J. Maaß J. -P. Boland W. Peiter E.. (2015). Systemic cytosolic Ca^2+^ elevation is activated upon wounding and herbivory in *Arabidopsis*. New Phytol. 207, 996–1004. 10.1111/nph.13493, PMID: 25996806

[ref169] Kim S. -Y. Sivaguru M. Stacey G. (2006). Extracellular ATP in plants. Visualization, localization, and analysis of physiological significance in growth and signaling. Plant Physiol. 142, 984–992. 10.1104/pp.106.085670, PMID: 16963521 PMC1630726

[ref170] Kimura S. Hunter K. Vaahtera L. Tran H. C. Citterico M. Vaattovaara A.. (2020). CRK2 and C-terminal phosphorylation of NADPH oxidase RBOHD regulate reactive oxygen species production in *Arabidopsis*. Plant Cell 32, 1063–1080. 10.1105/tpc.19.00525, PMID: 32034035 PMC7145479

[ref171] Klauser D. Desurmont G. A. Glauser G. Vallat A. Flury P. Boller T.. (2015). The *Arabidopsis* Pep-PEPR system is induced by herbivore feeding and contributes to JA-mediated plant defence against herbivory. J. Exp. Bot. 66, 5327–5336. 10.1093/jxb/erv250, PMID: 26034129 PMC4526914

[ref172] Klemenčič M. Funk C. (2018). Structural and functional diversity of caspase homologues in non-metazoan organisms. Protoplasma 255, 387–397. 10.1007/s00709-017-1145-5, PMID: 28744694 PMC5756287

[ref173] Knoblauch M. van Bel A. J. E. (1998). Sieve tubes in action. Plant Cell 10, 35–50. 10.1105/tpc.10.1.35

[ref175] Koo A. J. K. Howe G. A. (2009). The wound hormone jasmonate. Phytochemistry 70, 1571–1580. 10.1016/j.phytochem.2009.07.018, PMID: 19695649 PMC2784233

[ref176] Krol E. Mentzel T. Chinchilla D. Boller T. Felix G. Kemmerling B.. (2010). Perception of the *Arabidopsis* danger signal peptide 1 involves the pattern recognition receptor *At*PEPR1 and its close homologue *At*PEPR2. J. Biol. Chem. 285, 13471–13479. 10.1074/jbc.M109.097394, PMID: 20200150 PMC2859507

[ref177] Kudla J. Batistič O. Hashimoto K. (2010). Calcium signals: the lead currency of plant information processing. Plant Cell 22, 541–563. 10.1105/tpc.109.072686, PMID: 20354197 PMC2861448

[ref178] Kumari A. Chételat A. Nguyen C. T. Farmer E. E. (2019). *Arabidopsis* H^+^-ATPase AHA1 controls slow wave potential duration and wound-response jasmonate pathway activation. Proc. Natl. Acad. Sci. U. S. A. 116, 20226–20231. 10.1073/pnas.1907379116, PMID: 31527254 PMC6778210

[ref179] Kurenda A. Nguyen C. T. Chételat A. Stolz S. Farmer E. E. (2019). Insect-damaged *Arabidopsis* moves like wounded *Mimosa pudica*. Proc. Natl. Acad. Sci. U. S. A. 116, 26066–26071. 10.1073/pnas.1912386116, PMID: 31792188 PMC6926025

[ref180] Kuthanova A. Opatrny Z. Fischer L. (2008). Is internucleosomal DNA fragmentation an indicator of programmed death in plant cells? J. Exp. Bot. 59, 2233–2240. 10.1093/jxb/ern090, PMID: 18436542 PMC2413271

[ref181] Laluk K. Luo H. Chai M. Dhawan R. Lai Z. Mengiste T. (2011). Biochemical and genetic requirements for function of the immune response regulator BOTRYTIS-INDUCED KINASE1 in plant growth, ethylene signaling, and PAMP-triggered immunity in *Arabidopsis*. Plant Cell 23, 2831–2849. 10.1105/tpc.111.087122, PMID: 21862710 PMC3180795

[ref182] Larrieu A. Champion A. Legrand J. Lavenus J. Mast D. Brunoud G.. (2015). A fluorescent hormone biosensor reveals the dynamics of jasmonate signalling in plants. Nat. Commun. 6:6043. 10.1038/ncomms7043, PMID: 25592181 PMC4338584

[ref183] Lee M. W. Huffaker A. Crippen D. Robbins R. T. Goggin F. L. (2018). Plant elicitor peptides promote plant defences against nematodes in soybean. Mol. Plant Pathol. 19, 858–869. 10.1111/mpp.12570, PMID: 28600875 PMC6638146

[ref184] Lehmann P. Ammunét T. Barton M. Battisti A. Eigenbrode S. D. Jepsen J. U. (2020). Complex responses of global insect pests to climate warming. Front. Ecol. Environ. 10.1002/fee.2160 (in press).

[ref185] Leijon F. Melzer M. Zhou Q. Srivastava V. Bulone V. (2018). Proteomic analysis of plasmodesmata from populus cell suspension cultures in relation with callose biosynthesis. Front. Plant Sci. 9:1681. 10.3389/fpls.2018.01681, PMID: 30510561 PMC6252348

[ref186] Lenglet A. Jaślan D. Toyota M. Mueller M. Müller T. Schönknecht G.. (2017). Control of basal jasmonate signalling and defence through modulation of intracellular cation flux capacity. New Phytol. 216, 1161–1169. 10.1111/nph.14754, PMID: 28885692

[ref187] León J. Rojo E. Sánchez-Serrano J. J. (2001). Wound signalling in plants. J. Exp. Bot. 52, 1–9. 10.1093/jexbot/52.354.1, PMID: 11181708

[ref188] Leone M. Keller M. M. Cerrudo I. Ballaré C. L. (2014). To grow or defend? Low red:far-red ratios reduce jasmonate sensitivity in *Arabidopsis* seedlings by promoting DELLA degradation and increasing JAZ10 stability. New Phytol. 204, 355–367. 10.1111/nph.12971, PMID: 25103816

[ref189] Levy M. Wang Q. Kaspi R. Parrella M. P. Abel S. (2005). *Arabidopsis* IQD1, a novel calmodulin-binding nuclear protein, stimulates glucosinolate accumulation and plant defense. Plant J. 43, 79–96. 10.1111/j.1365-313X.2005.02435.x, PMID: 15960618

[ref190] Lew T. T. S. Koman V. B. Silmore K. S. Seo J. S. Gordiichuk P. Kwak S. -Y.. (2020). Real-time detection of wound-induced H_2_O_2_ signalling waves in plants with optical nanosensors. Nat. Plants 6, 404–415. 10.1038/s41477-020-0632-4, PMID: 32296141

[ref191] L’Haridon F. Besson-Bard A. Binda M. Serrano M. Abou-Mansour E. Balet F.. (2011). A permeable cuticle is associated with the release of reactive oxygen species and induction of innate immunity. PLoS Pathog. 7:e1002148. 10.1371/journal.ppat.1002148, PMID: 21829351 PMC3145797

[ref192] Li G. Bartram S. Guo H. Mithöfer A. Kunert M. Boland W. (2019). SpitWorm, a herbivorous robot: mechanical leaf wounding with simultaneous application of salivary components. Plan. Theory 8:318. 10.3390/plants8090318, PMID: 31480435 PMC6784092

[ref193] Li S. Han X. Yang L. Deng X. Wu H. Zhang M.. (2018). Mitogen-activated protein kinases and calcium-dependent protein kinases are involved in wounding-induced ethylene biosynthesis in *Arabidopsis thaliana*. Plant Cell Environ. 41, 134–147. 10.1111/pce.12984, PMID: 28543054

[ref194] Li Q. Wang C. Mou Z. (2020). Perception of damaged self in plants. Plant Physiol. 182, 1545–1565. 10.1104/PP.19.01242, PMID: 31907298 PMC7140957

[ref195] Li T. Yan A. Bhatia N. Altinok A. Afik E. Durand-Smet P.. (2019). Calcium signals are necessary to establish auxin transporter polarity in a plant stem cell niche. Nat. Commun. 10:726. 10.1038/s41467-019-08575-6, PMID: 30760714 PMC6374474

[ref196] Li Q. Zheng J. Li S. Huang G. Skilling S. J. Wang L.. (2017). Transporter-mediated nuclear entry of jasmonoyl-isoleucine is essential for jasmonate signaling. Mol. Plant 10, 695–708. 10.1016/j.molp.2017.01.010, PMID: 28179150

[ref197] Lipetz J. (1970). Wound-healing in higher plants. Int. Rev. Cytol. 27, 1–28. 10.1016/S0074-7696(08)61244-9

[ref198] Liu J. Sheng L. Xu Y. Li J. Yang Z. Huang H.. (2014). *WOX11* and *12* are involved in the first-step cell fate transition during de novo root organogenesis in *Arabidopsis*. Plant Cell 26, 1081–1093. 10.1105/tpc.114.122887, PMID: 24642937 PMC4001370

[ref199] Locci F. Benedetti M. Pontiggia D. Citterico M. Caprari C. Mattei B.. (2019). An *Arabidopsis* berberine bridge enzyme-like protein specifically oxidizes cellulose oligomers and plays a role in immunity. Plant J. 98, 540–554. 10.1111/tpj.14237, PMID: 30664296

[ref200] Lori M. van Verk M. C. Hander T. Schatowitz H. Klauser D. Flury P.. (2015). Evolutionary divergence of the plant elicitor peptides (Peps) and their receptors: interfamily incompatibility of perception but compatibility of downstream signalling. J. Exp. Bot. 66, 5315–5325. 10.1093/jxb/erv236, PMID: 26002971 PMC4526913

[ref201] Lotze M. T. Tracey K. J. (2005). High-mobility group box 1 protein (HMGB1): nuclear weapon in the immune arsenal. Nat. Rev. Immunol. 5, 331–342. 10.1038/nri1594, PMID: 15803152

[ref202] Lu L. Yang Y. Zhang H. Sun D. Li Z. Guo Q.. (2021). Oligogalacturonide-accelerated healing of mechanical wounding in tomato fruit requires calcium-dependent systemic acquired resistance. Food Chem. 337:127992. 10.1016/j.foodchem.2020.127992, PMID: 32920270

[ref203] Lulai E. C. Campbell L. G. Fugate K. K. McCue K. F. (2016). Biological differences that distinguish the 2 major stages of wound healing in potato tubers. Plant Signal. Behav. 11:e1256531. 10.1080/15592324.2016.1256531, PMID: 27831001 PMC5225934

[ref204] Lulai E. C. Corsini D. L. (1998). Differential deposition of suberin phenolic and aliphatic domains and their roles in resistance to infection during potato tuber (*Solanum tuberosum* L.) wound-healing. Physiol. Mol. Plant Pathol. 53, 209–222. 10.1006/pmpp.1998.0179

[ref205] Maffei M. Bossi S. Spiteller D. Mithöfer A. Boland W. (2004). Effects of feeding *Spodoptera littoralis* on Lima bean leaves. I. Membrane potentials, intracellular calcium variations, oral secretions, and regurgitate components. Plant Physiol. 134, 1752–1762. 10.1104/pp.103.034165, PMID: 15051862 PMC419848

[ref206] Maffei M. E. Mithöfer A. Arimura G. -I. Uchtenhagen H. Bossi S. Bertea C. M.. (2006). Effects of feeding *Spodoptera littoralis* on Lima bean leaves. III. Membrane depolarization and involvement of hydrogen peroxide. Plant Physiol. 140, 1022–1035. 10.1104/pp.105.071993, PMID: 16443697 PMC1400574

[ref207] Maffei M. E. Mithöfer A. Boland W. (2007a). Before gene expression: early events in plant-insect interaction. Trends Plant Sci. 12, 310–316. 10.1016/j.tplants.2007.06.001, PMID: 17596996

[ref384] Maffei M. E. Mithöfer A. Boland W. (2007b). Insects feeding on plants: rapid signals and responses preceding the induction of phytochemical release. Phytochemistry 68, 2946–2959. 10.1016/j.phytochem.2007.07.016, PMID: 17825328

[ref209] Major I. T. Guo Q. Zhai J. Kapali G. Kramer D. M. Howe G. A. (2020). A phytochrome B-independent pathway restricts growth at high levels of jasmonate defense. Plant Physiol. 183, 733–749. 10.1104/pp.19.01335, PMID: 32245790 PMC7271779

[ref210] Major I. T. Yoshida Y. Campos M. L. Kapali G. Xin X. -F. Sugimoto K.. (2017). Regulation of growth-defense balance by the JASMONATE ZIM-DOMAIN (JAZ)-MYC transcriptional module. New Phytol. 215, 1533–1547. 10.1111/nph.14638, PMID: 28649719 PMC5542871

[ref211] Malone M. (1992). Kinetics of wound-induced hydraulic signals and variation potentials in wheat seedlings. Planta 187, 505–510. 10.1007/BF00199969, PMID: 24178145

[ref212] Malone M. Stanković B. (1991). Surface potentials and hydraulic signals in wheat leaves following localized wounding by heat. Plant Cell Environ. 14, 431–436. 10.1111/j.1365-3040.1991.tb00953.x

[ref213] Marhava P. Hoermayer L. Yoshida S. Marhavý P. Benková E. Friml J. (2019). Re-activation of stem cell pathways for pattern restoration in plant wound healing. Cell 177, 957–969. 10.1016/j.cell.2019.04.015, PMID: 31051107 PMC6506278

[ref214] Marhavý P. Kurenda A. Siddique S. Dénervaud Tendon V. Zhou F. Holbein J.. (2019). Single-cell damage elicits regional, nematode-restricting ethylene responses in roots. EMBO J. 38:e100972. 10.15252/embj.2018100972, PMID: 31061171 PMC6518030

[ref215] Marichal T. Ohata K. Bedoret D. Mesnil C. Sabatel C. Kobiyama K.. (2011). DNA released from dying host cells mediates aluminum adjuvant activity. Nat. Med. 17, 996–1002. 10.1038/nm.2403, PMID: 21765404

[ref216] Matosevich R. Cohen I. Gil-Yarom N. Modrego A. Friedlander-Shani L. Verna C.. (2020). Local auxin biosynthesis is required for root regeneration after wounding. Nat. Plants 6, 1020–1030. 10.1038/s41477-020-0737-9, PMID: 32747761

[ref217] Matsuoka D. Nanmori T. Sato K. -I. Fukami Y. Kikkawa U. Yasuda T. (2002). Activation of AtMEK1, an *Arabidopsis* mitogen-activated protein kinase kinase, *in vitro* and *in vivo*: analysis of active mutants expressed in *E. coli* and generation of the active form in stress response in seedlings. Plant J. 29, 637–647. 10.1046/j.0960-7412.2001.01246.x, PMID: 11874576

[ref218] Matsuoka K. Sugawara E. Aoki R. Takuma K. Terao-Morita M. Satoh S.. (2016). Differential cellular control by cotyledon-derived phytohormones involved in graft reunion of *Arabidopsis* hypocotyls. Plant Cell Physiol. 57, 2620–2631. 10.1093/pcp/pcw177, PMID: 27986917

[ref219] Matzinger P. (1994). Tolerance, danger, and the extended family. Annu. Rev. Immunol. 12, 991–1045. 10.1146/annurev.iy.12.040194.005015, PMID: 8011301

[ref220] McConnell E. W. Berg P. Westlake T. J. Wilson K. M. Popescu G. V. Hicks L. M.. (2019). Proteome-wide analysis of cysteine reactivity during effector-triggered immunity. Plant Physiol. 179, 1248–1264. 10.1104/pp.18.01194, PMID: 30510037 PMC6446758

[ref221] McGurl B. Pearce G. Orozco-Cardenas M. Ryan C. A. (1992). Structure, expression, and antisense inhibition of the systemin precursor gene. Science 255, 1570–1573. 10.1126/science.1549783, PMID: 1549783

[ref222] Medina-Castellanos E. Esquivel-Naranjo E. U. Heil M. Herrera-Estrella A. (2014). Extracellular ATP activates MAPK and ROS signaling during injury response in the fungus *Trichoderma atroviride*. Front. Plant Sci. 5:659. 10.3389/fpls.2014.00659, PMID: 25484887 PMC4240048

[ref223] Meena M. K. Prajapati R. Krishna D. Divakaran K. Pandey Y. Reichelt M.. (2019). The Ca^2+^ channel CNGC19 regulates *Arabidopsis* defense against Spodoptera herbivory. Plant Cell 31, 1539–1562. 10.1105/tpc.19.00057, PMID: 31076540 PMC6635850

[ref224] Melnyk C. W. Gabel A. Hardcastle T. J. Robinson S. Miyashima S. Grosse I.. (2018). Transcriptome dynamics at *Arabidopsis* graft junctions reveal an intertissue recognition mechanism that activates vascular regeneration. Proc. Natl. Acad. Sci. U. S. A. 115, E2447–E2456. 10.1073/pnas.1718263115, PMID: 29440499 PMC5878008

[ref225] Melnyk C. W. Schuster C. Leyser O. Meyerowitz E. M. (2015). A developmental framework for graft formation and vascular reconnection in *Arabidopsis thaliana*. Curr. Biol. 25, 1306–1318. 10.1016/j.cub.2015.03.032, PMID: 25891401 PMC4798781

[ref226] Mhamdi A. (2019). The immune redoxome: effector-triggered immunity switches cysteine oxidation profiles. Plant Physiol. 179, 1196–1197. 10.1104/pp.19.00207, PMID: 30940734 PMC6446770

[ref227] Mielke S. Gasperini D. (2019). Interplay between plant cell walls and jasmonate production. Plant Cell Physiol. 60, 2629–2637. 10.1093/pcp/pcz119, PMID: 31241137

[ref228] Mignolet-Spruyt L. Xu E. Idänheimo N. Hoeberichts F. A. Mühlenbock P. Brosché M.. (2016). Spreading the news: subcellular and organellar reactive oxygen species production and signalling. J. Exp. Bot. 67, 3831–3844. 10.1093/jxb/erw080, PMID: 26976816

[ref229] Miller G. Schlauch K. Tam R. Cortes D. Torres M. A. Shulaev V.. (2009). The plant NADPH oxidase RBOHD mediates rapid systemic signaling in response to diverse stimuli. Sci. Signal. 2:ra45. 10.1126/scisignal.2000448, PMID: 19690331

[ref230] Minibayeva F. V. Gordon L. K. Kolesnikov O. P. Chasov A. V. (2001). Role of extracellular peroxidase in the superoxide production by wheat root cells. Protoplasma 217, 125–128. 10.1007/BF01289421, PMID: 11732329

[ref231] Minina E. A. Staal J. Alvarez V. E. Berges J. A. Berman-Frank I. Beyaert R.. (2020). Classification and nomenclature of metacaspases and paracaspases: no more confusion with caspases. Mol. Cell 77, 927–929. 10.1016/j.molcel.2019.12.020, PMID: 32142688 PMC7325697

[ref232] Mithöfer A. Boland W. (2012). Plant defense against herbivores: chemical aspects. Annu. Rev. Plant Biol. 63, 431–450. 10.1146/annurev-arplant-042110-103854, PMID: 22404468

[ref233] Mithöfer A. Wanner G. Boland W. (2005). Effects of feeding *Spodoptera littoralis* on Lima bean leaves. II. Continuous mechanical wounding resembling insect feeding is sufficient to elicit herbivory-related volatile emission. Plant Physiol. 137, 1160–1168. 10.1104/pp.104.054460, PMID: 15728342 PMC1065415

[ref234] Moloshok T. Pearce G. Ryan C. A. (1992). Oligouronide signaling of proteinase inhibitor genes in plants: structure-activity relationships of di- and trigalacturonic acids and their derivatives. Arch. Biochem. Biophys. 294, 731–734. 10.1016/0003-9861(92)90748-L, PMID: 1567229

[ref235] Mosblech A. Thurow C. Gatz C. Feussner I. Heilmann I. (2011). Jasmonic acid perception by COI1 involves inositol polyphosphates in *Arabidopsis thaliana*. Plant J. 65, 949–957. 10.1111/j.1365-313X.2011.04480.x, PMID: 21205029

[ref236] Mousavi S. A. R. Chauvin A. Pascaud F. Kellenberger S. Farmer E. E. (2013). *GLUTAMATE RECEPTOR-LIKE* genes mediate leaf-to-leaf wound signalling. Nature 500, 422–426. 10.1038/nature12478, PMID: 23969459

[ref377] Moyen C. Hammond-Kosack K. E. Jones J. Knight M. R. Johannes E. (1998). Systemin triggers an increase of cytoplasmic calcium in tomato mesophyll cells: Ca^2+^ mobilization from intra-and extracellular compartments. Plant Cell Environ. 21, 1101–1111. 10.1046/j.1365-3040.1998.00378.x

[ref237] Mullendore D. L. Windt C. W. Van As H. Knoblauch M. (2010). Sieve tube geometry in relation to phloem flow. Plant Cell 22, 579–593. 10.1105/tpc.109.070094, PMID: 20354199 PMC2861446

[ref238] Nakaminami K. Okamoto M. Higuchi-Takeuchi M. Yoshizumi T. Yamaguchi Y. Fukao Y.. (2018). AtPep3 is a hormone-like peptide that plays a role in the salinity stress tolerance of plants. Proc. Natl. Acad. Sci. U. S. A. 115, 5810–5815. 10.1073/pnas.1719491115, PMID: 29760074 PMC5984501

[ref378] Narváez-Vásquez J. Florin-Christensen J. Ryan C. A. (1999). Positional specificity of a phospholipase A activity induced by wounding, systemin, and oligosaccharide elicitors in tomato leaves. Plant Cell 11, 2249–2260. 10.1105/tpc.11.11.2249, PMID: 10559447 PMC144127

[ref239] Narváez-Vásquez J. Ryan C. A. (2004). The cellular localization of prosystemin: a functional role for phloem parenchyma in systemic wound signaling. Planta 218, 360–369. 10.1007/s00425-003-1115-3, PMID: 14534786

[ref240] Nguyen C. T. Kurenda A. Stolz S. Chételat A. Farmer E. E. (2018). Identification of cell populations necessary for leaf-toleaf electrical signaling in a wounded plant. Proc. Natl. Acad. Sci. U. S. A. 115, 10178–10183. 10.1073/pnas.1807049115, PMID: 30228123 PMC6176584

[ref241] Ninkovic V. Rensing M. Dahlin I. Markovic D. (2019). Who is my neighbor? Volatile cues in plant interactions. Plant Signal. Behav. 9:1634993. 10.1080/15592324.2019.1634993, PMID: 31267830 PMC6768235

[ref242] Notaguchi M. Kurotani K. -i. Sato Y. Tabata R. Kawakatsu Y. Okayasu K. (2020). Cell-cell adhesion in plant grafting is facilitated by β-1,4-glucanases. Science 369, 698–702. 10.1126/science.abc3710, PMID: 32764072

[ref243] Nožková V. Šmíd P. Horváth P. Hrabovský M. Ilík P. (2018). Non-invasive monitoring of hydraulic surge propagation in a wounded tobacco plant. Plant Methods 14:38. 10.1186/s13007-018-0307-6, PMID: 29849744 PMC5968581

[ref244] Nühse T. S. (2012). Cell wall integrity signaling and innate immunity in plants. Front. Plant Sci. 3:280. 10.3389/fpls.2012.00280, PMID: 23248636 PMC3518785

[ref245] Ogita N. Okushima Y. Tokizawa M. Yamamoto Y. Y. Tanaka M. Seki M.. (2018). Identifying the target genes of SUPPRESSOR OF GAMMA RESPONSE 1, a master transcription factor controlling DNA damage response in *Arabidopsis*. Plant J. 94, 439–453. 10.1111/tpj.13866, PMID: 29430765

[ref246] Orozco-Cárdenas M. L. Narváez-Vásquez J. Ryan C. A. (2001). Hydrogen peroxide acts as a second messenger for the induction of defense genes in tomato plants in response to wounding, systemin, and methyl jasmonate. Plant Cell 13, 179–191. 10.1105/tpc.13.1.179, PMID: 11158538 PMC102208

[ref247] Ortiz-Masia D. Perez-Amador M. A. Carbonell J. Marcote M. J. (2007). Diverse stress signals activate the C1 subgroup MAP kinases of *Arabidopsis*. FEBS Lett. 581, 1834–1840. 10.1016/j.febslet.2007.03.075, PMID: 17433310

[ref248] Ortiz-Morea F. A. Savatin D. V. Dejonghe W. Kumar R. Luo Y. Adamowski M.. (2016). Danger-associated peptide signaling in *Arabidopsis* requires clathrin. Proc. Natl. Acad. Sci. U. S. A. 113, 11028–11033. 10.1073/pnas.1605588113, PMID: 27651494 PMC5047203

[ref249] Pattyn J. Vaughan-Hirsch J. Van de Poel B. (2020). The regulation of ethylene biosynthesis: a complex multilevel control circuitry. New Phytol. 10.1111/nph.16873, PMID: [Epub ahead of print] 32790878 PMC7820975

[ref250] Pauwels L. Morreel K. De Witte E. Lammertyn F. Van Montagu M. Boerjan W.. (2008). Mapping methyl jasmonate-mediated transcriptional reprogramming of metabolism and cell cycle progression in cultured *Arabidopsis* cells. Proc. Natl. Acad. Sci. U. S. A. 105, 1380–1385. 10.1073/pnas.0711203105, PMID: 18216250 PMC2234147

[ref251] Pearce G. (2011). Systemin, hydroxyproline-rich systemin and the induction of protease inhibitors. Curr. Protein Pept. Sci. 12, 399–405. 10.2174/138920311796391106, PMID: 21418016

[ref252] Pearce G. Moura D. S. Stratmann J. Ryan C. A. (2001). Production of multiple plant hormones from a single polyprotein precursor. Nature 411, 817–820. 10.1038/35081107, PMID: 11459063

[ref253] Pearce G. Siems W. F. Bhattacharya R. Chen Y.-C. Ryan C. A. (2007). Three hydroxyproline-rich glycopeptides derived from a single petunia polyprotein precursor activate *defensin I*, a pathogen defense response gene. J. Biol. Chem. 282, 17777–17784. 10.1074/jbc.M701543200, PMID: 17449475

[ref254] Pearce G. Strydom D. Johnson S. Ryan C. A. (1991). A polypeptide from tomato leaves induces wound-inducible proteinase inhibitor proteins. Science 253, 895–897. 10.1126/science.253.5022.895, PMID: 17751827

[ref255] Pedersen D. S. Merkle T. Marktl B. Lildballe D. L. Antosch M. Bergmann T.. (2010). Nucleocytoplasmic distribution of the *Arabidopsis* chromatin-associated HMGB2/3 and HMGB4 proteins. Plant Physiol. 154, 1831–1841. 10.1104/pp.110.163055, PMID: 20940346 PMC2996034

[ref256] Peña-Cortés H. Fisahn J. Willmitzer L. (1995). Signals involved in wound-induced proteinase inhibitor II gene expression in tomato and potato plants. Proc. Natl. Acad. Sci. U. S. A. 92, 4106–4113. 10.1073/pnas.92.10.4106, PMID: 11607535 PMC41894

[ref257] Pēna-Cortés H. Sánchez-Serrano J. J. Mertens R. Willmitzer L. Prat S. (1989). Abscisic acid is involved in the wound-induced expression of the proteinase inhibitor II gene in potato and tomato. Proc. Natl. Acad. Sci. U. S. A. 86, 9851–9855. 10.1073/pnas.86.24.9851, PMID: 16594093 PMC298600

[ref258] Pham A. Q. Cho S. -H. Nguyen C. T. Stacey G. (2020). *Arabidopsis* lectin receptor kinase P2K2 is a second plant receptor for extracellular ATP and contributes to innate immunity. Plant Physiol. 183, 1364–1375. 10.1104/pp.19.01265, PMID: 32345768 PMC7333714

[ref259] Pottecher J. Meyer A. Ferreira Wenceslau C. Timmermans K. Hauser C. J. Land W. G. (2019). Editorial: trauma-induced, DAMP-mediated remote organ injury, and immunosuppression in the acutely ill patient. Front. Immunol. 10:1971. 10.3389/fimmu.2019.01971, PMID: 31481961 PMC6710339

[ref379] O’Donnell P. J. Calvert C. Atzorn R. Wasternack C. Leyser H. M. O. Bowles D. J. (1996). Ethylene as a signal mediating the wound response of tomato plants. Science 274, 1914–1917. 10.1126/science.274.5294.1914, PMID: 8943205

[ref380] Orozco-Cardenas M. Ryan C. A. (1999). Hydrogen peroxide is generated systemically in plant leaves by wounding and systemin via the octadecanoid pathway. Proc. Natl. Acad. Sci. U. S. A. 96, 6553–6557. 10.1073/pnas.96.11.6553, PMID: 10339626 PMC26920

[ref260] Qi Z. Stephens N. R. Spalding E. P. (2006). Calcium entry mediated by GLR3.3, an *Arabidopsis* glutamate receptor with a broad agonist profile. Plant Physiol. 142, 963–971. 10.1104/pp.106.088989, PMID: 17012403 PMC1630757

[ref381] Ranf S. Eschen-Lippold L. Pecher P. Lee J. Scheel D. (2011). Interplay between calcium signalling and early signalling elements during defence responses to microbe- or damage-associated molecular patterns. Plant J. 68, 100–113. 10.1111/j.1365-313X.2011.04671.x, PMID: 21668535

[ref261] Rasul S. Dubreuil-Maurizi C. Lamotte O. Koen E. Poinssot B. Alcaraz G.. (2012). Nitric oxide production mediates oligogalacturonide-triggered immunity and resistance to Botrytis cinerea in *Arabidopsis thaliana*. Plant Cell Environ. 35, 1483–1499. 10.1111/j.1365-3040.2012.02505.x, PMID: 22394204

[ref262] Razzell W. Evans I. R. Martin P. Wood W. (2013). Calcium flashes orchestrate the wound inflammatory response through DUOX activation and hydrogen peroxide release. Curr. Biol. 23, 424–429. 10.1016/j.cub.2013.01.058, PMID: 23394834 PMC3629559

[ref263] Ren F. Lu Y. -T. (2006). Overexpression of tobacco hydroxyproline-rich glycopeptide systemin precursor A gene in transgenic tobacco enhances resistance against *Helicoverpa armigera* larvae. Plant Sci. 171, 286–292. 10.1016/j.plantsci.2006.04.001

[ref264] Ricca U. (1916). Soluzione d’un problema di fisiologia: La propagazione di stimolo nella “Mimosa.” Nuovo Giorn. Bot. Ital. 23, 51–170.

[ref265] Ross A. Yamada K. Hiruma K. Yamashita-Yamada M. Lu X. Takano Y.. (2014). The *Arabidopsis* PEPR pathway couples local and systemic plant immunity. EMBO J. 33, 62–75. 10.1002/embj.201284303, PMID: 24357608 PMC3990683

[ref266] Routier-Kierzkowska A. -L. Weber A. Kochova P. Felekis D. Nelson B. J. Kuhlemeier C.. (2012). Cellular force microscopy for *in vivo* measurements of plant tissue mechanics. Plant Physiol. 158, 1514–1522. 10.1104/pp.111.191460, PMID: 22353572 PMC3343728

[ref267] Roux S. J. Clark G. (2019). “Extracellular ATP signaling in animals and plants: comparison and contrast” in Sensory biology of plants. ed. Sopory S. (Singapore: Springer), 389–409.

[ref268] Ryan C. A. Jagendorf A. (1995). Self-defense by plants. Proc. Natl. Acad. Sci. U. S. A. 92:4075. 10.1073/pnas.92.10.4075, PMID: 7753773 PMC41888

[ref269] Ryan C. A. Pearce G. (2003). Systemins: a functionally defined family of peptide signals that regulate defensive genes in Solanaceae species. Proc. Natl. Acad. Sci. U. S. A. 100, 14577–14580. 10.1073/pnas.1934788100, PMID: 12949264 PMC304121

[ref270] Ryerson D. E. Heath M. C. (1996). Cleavage of nuclear DNA into oligonucleosomal fragments during cell death induced by fungal infection or by abiotic treatments. Plant Cell 8, 393–402. 10.1105/tpc.8.3.393, PMID: 12239388 PMC161108

[ref271] Saltveit M. E. (2016). The three responses of plant tissue to wounding. Acta Hortic. 1141, 13–20. 10.17660/ActaHortic.2016.1141.2

[ref272] Salvador-Recatalà V. (2016). New roles for the *GLUTAMATE RECEPTOR-LIKE 3.3*, *3.5*, and *3.6* genes as on/off switches of wound-induced systemic electrical signals. Plant Signal. Behav. 11:e1161879. 10.1080/15592324.2016.1161879, PMID: 26966923 PMC4883974

[ref273] Salvador-Recatalà V. Tjallingii W. F. Farmer E. E. (2014). Real-time, *in vivo* intracellular recordings of caterpillar-induced depolarization waves in sieve elements using aphid electrodes. New Phytol. 203, 674–684. 10.1111/nph.12807, PMID: 24716546

[ref274] Santamaria M. E. Martínez M. Cambra I. Grbic V. Diaz I. (2013). Understanding plant defence responses against herbivore attacks: an essential first step towards the development of sustainable resistance against pests. Transgenic Res. 22, 697–708. 10.1007/s11248-013-9725-4, PMID: 23793555

[ref275] Savatin D. V. Gramegna G. Modesti V. Cervone F. (2014). Wounding in the plant tissue: the defense of a dangerous passage. Front. Plant Sci. 5:470. 10.3389/fpls.2014.00470, PMID: 25278948 PMC4165286

[ref276] Schaller A. Stintzi A. (2009). Enzymes in jasmonate biosynthesis—structure, function, regulation. Phytochemistry 70, 1532–1538. 10.1016/j.phytochem.2009.07.032, PMID: 19703696

[ref382] Schardon K. Hohl M. Graff L. Pfannstiel J. Schulze W. Stintzi A.. (2016). Precursor processing for plant peptide hormone maturation by subtilisin-like serine proteinases. Science 354, 1594–1597. 10.1126/science.aai8550, PMID: 27940581

[ref277] Schmelz E. A. (2015). Impacts of insect oral secretions on defoliation-induced plant defense. Curr. Opin. Insect Sci. 9, 7–15. 10.1016/j.cois.2015.04.002, PMID: 32846712

[ref278] Schmelz E. A. Carroll M. J. LeClere S. Phipps S. M. Meredith J. Chourey P. S.. (2006). Fragments of ATP synthase mediate plant perception of insect attack. Proc. Natl. Acad. Sci. U. S. A. 103, 8894–8899. 10.1073/pnas.0602328103, PMID: 16720701 PMC1482674

[ref279] Schmelz E. A. LeClere S. Carroll M. J. Alborn H. T. Teal P. E. A. (2007). Cowpea chloroplastic ATP synthase is the source of multiple plant defense elicitors during insect herbivory. Plant Physiol. 144, 793–805. 10.1104/pp.107.097154, PMID: 17369425 PMC1914193

[ref280] Scholz S. S. Vadassery J. Heyer M. Reichelt M. Bender K. W. Snedden W. A.. (2014). Mutation of the *Arabidopsis* calmodulin-like protein CML37 deregulates the jasmonate pathway and enhances susceptibility to herbivory. Mol. Plant 7, 1712–1726. 10.1093/mp/ssu102, PMID: 25267731

[ref281] Schulze A. Zimmer M. Mielke S. Stellmach H. Melnyk C. W. Hause B.. (2019). Wound-induced shoot-to-root relocation of JA-Ile precursors coordinates *Arabidopsis* growth. Mol. Plant 12, 1383–1394. 10.1016/j.molp.2019.05.013, PMID: 31181337

[ref282] Schweighofer A. Kazanaviciute V. Scheikl E. Teige M. Doczi R. Hirt H.. (2007). The PP2C-type phosphatase AP2C1, which negatively regulates MPK4 and MPK6, modulates innate immunity, jasmonic acid, and ethylene levels in *Arabidopsis*. Plant Cell 19, 2213–2224. 10.1105/tpc.106.049585, PMID: 17630279 PMC1955703

[ref283] Sena G. Wang X. Liu H.-Y. Hofhuis H. Birnbaum K. D. (2009). Organ regeneration does not require a functional stem cell niche in plants. Nature 457, 1150–1153. 10.1038/nature07597, PMID: 19182776 PMC2649681

[ref284] Seo S. Okamoto M. Seto H. Ishizuka K. Sano H. Ohashi Y. (1995). Tobacco MAP kinase: a possible mediator in wound signal transduction pathways. Science 270, 1988–1992. 10.1126/science.270.5244.1988, PMID: 8533090

[ref285] Seo S. Sano H. Ohashi Y. (1999). Jasmonate-based wound signal transduction requires activation of WIPK, a tobacco mitogen-activated protein kinase. Plant Cell 11, 289–298. 10.1105/tpc.11.2.289, PMID: 9927645 PMC144162

[ref286] Shanmukhan A. P. Mathew M. M. Radhakrishnan D. Aiyaz M. Prasad K. (2020). Regrowing the damaged or lost body parts. Curr. Opin. Plant Biol. 53, 117–127. 10.1016/j.pbi.2019.12.007, PMID: 31962252

[ref287] Shannon E. K. Stevens A. Edrington W. Zhao Y. Jayasinghe A. K. Page-McCaw A.. (2017). Multiple mechanisms drive calcium signal dynamics around laser-induced epithelial wounds. Biophys. J. 113, 1623–1635. 10.1016/j.bpj.2017.07.022, PMID: 28978452 PMC5627067

[ref288] Shao Q. Gao Q. Lhamo D. Zhang H. Luan S. (2020). Two glutamate- and pH-regulated Ca^2+^ channels are required for systemic wound signaling in *Arabidopsis*. Sci. Signal. 13:eaba1453. 10.1126/scisignal.aba1453, PMID: 32665412

[ref289] Sheard L. B. Tan X. Mao H. Withers J. Ben-Nissan G. Hinds T. R.. (2010). Jasmonate perception by inositol-phosphate-potentiated COI1-JAZ co-receptor. Nature 468, 400–405. 10.1038/nature09430, PMID: 20927106 PMC2988090

[ref290] Shen W. Liu J. Li J. F. (2019). Type-II metacaspases mediate the processing of plant elicitor peptides in *Arabidopsis*. Mol. Plant 12, 1524–1533. 10.1016/j.molp.2019.08.003, PMID: 31454707

[ref291] Shinya T. Yasuda S. Hyodo K. Tani R. Hojo Y. Fujiwara Y.. (2018). Integration of danger peptide signals with herbivore-associated molecular pattern signaling amplifies anti-herbivore defense responses in rice. Plant J. 94, 626–637. 10.1111/tpj.13883, PMID: 29513388

[ref292] Si T. Wang X. Zhao C. Huang M. Cai J. Zhou Q.. (2018). The role of hydrogen peroxide in mediating the mechanical wounding-induced freezing tolerance in wheat. Front. Plant Sci. 9:327. 10.3389/fpls.2018.00327, PMID: 29593774 PMC5861560

[ref293] Song J. Durrant W. E. Wang S. Yan S. Tan E. H. Dong X. (2011). DNA repair proteins are directly involved in regulation of gene expression during plant immune response. Cell Host Microbe 9, 115–124. 10.1016/j.chom.2011.01.011, PMID: 21320694

[ref294] Song C. J. Steinebrunner I. Wang X. Stout S. C. Roux S. J. (2006). Extracellular ATP induces the accumulation of superoxide *via* NADPH oxidases in *Arabidopsis*. Plant Physiol. 140, 1222–1232. 10.1104/pp.105.073072, PMID: 16428598 PMC1435826

[ref295] Sözen C. Schenk S. T. Boudsocq M. Chardin C. Almeida-Trapp M. Krapp A.. (2020). Wounding and insect feeding trigger two independent MAPK pathways with distinct regulation and kinetics. Plant Cell 32, 1988–2003. 10.1105/tpc.19.00917, PMID: 32265268 PMC7268812

[ref296] Stahlberg R. (2006). Historical overview on plant neurobiology. Plant Signal. Behav. 1, 6–8. 10.4161/psb.1.1.2278, PMID: 19521469 PMC2633693

[ref297] Stahlberg R. Cleland R. E. Van Volkenburgh E. (2006). “Slow wave potentials—a propagating electrical signal unique to higher plants” in Communication in plants: Neuronal aspects of plant life. eds. Baluška F. Mancuso S. Volkmann D. (Berlin: Springer), 291–308.

[ref298] Stahlberg R. Cosgrove D. J. (1992). Rapid alterations in growth rate and electrical potentials upon stem excision in pea seedlings. Planta 187, 523–531. 10.1007/BF00199972, PMID: 11538115

[ref299] Stahlberg R. Cosgrove D. J. (1995). Comparison of electric and growth responses to excision in cucumber and pea seedlings. II. Long-distance effects are caused by the release of xylem pressure. Plant Cell Environ. 18, 33–41. 10.1111/j.1365-3040.1995.tb00541.x, PMID: 11541062

[ref300] Stahlberg R. Cosgrove D. J. (1996). Induction and ionic basis of slow wave potentials in seedlings of *Pisum sativum* L. Planta 200, 416–425. 10.1007/BF00231397, PMID: 11541124

[ref301] Steinbrenner A. D. Muñoz-Amatriaín M. Aguilar Venegas J. M. Lo S. Shi D. Holton N. (2019). A receptor for herbivore-associated molecular patterns mediates plant immunity. bioRxiv [Preprint]. 10.1101/679803 PMC7733821 33229576

[ref383] Stratmann J. W. Ryan C. A. (1997). Myelin basic protein kinase activity in tomato leaves is induced systemically by wounding and increases in response to systemin and oligosaccharide elicitors. Proc. Natl. Acad. Sci. U. S. A. 94, 11085–11089. 10.1073/pnas.94.20.11085, PMID: 9380763 PMC23618

[ref302] Stührwohldt N. Schaller A. (2019). Regulation of plant peptide hormones and growth factors by post-translational modification. Plant Biol. Suppl. 1, 49–63. 10.1111/plb.12881, PMID: 30047205

[ref303] Sun J. Zhang X. Deng S. Zhang C. Wang M. Ding M.. (2012). Extracellular ATP signaling is mediated by H_2_O_2_ and cytosolic Ca^2+^ in the salt response of *Populus euphratica* cells. PLoS One 7:e53136. 10.1371/journal.pone.0053136, PMID: 23285259 PMC3532164

[ref304] Surova O. Zhivotovsky B. (2013). Various modes of cell death induced by DNA damage. Oncogene 32, 3789–3797. 10.1038/onc.2012.556, PMID: 23208502

[ref305] Suza W. P. Staswick P. E. (2008). The role of JAR1 in Jasmonoyl-L-isoleucine production during *Arabidopsis* wound response. Planta 227, 1221–1232. 10.1007/s00425-008-0694-4, PMID: 18247047

[ref306] Suzuki N. Miller G. Morales J. Shulaev V. Torres M. A. Mittler R. (2011). Respiratory burst oxidases: the engines of ROS signaling. Curr. Opin. Plant Biol. 14, 691–699. 10.1016/j.pbi.2011.07.014, PMID: 21862390

[ref307] Suzuki N. Mittler R. (2012). Reactive oxygen species-dependent wound responses in animals and plants. Free Radic. Biol. Med. 53, 2269–2276. 10.1016/j.freeradbiomed.2012.10.538, PMID: 23085520

[ref308] Szechyńska-Hebda M. Lewandowska M. Karpiński S. (2017). Electrical signaling, photosynthesis and systemic acquired acclimation. Front. Physiol. 8:684. 10.3389/fphys.2017.00684, PMID: 28959209 PMC5603676

[ref309] Takahashi F. Mizoguchi T. Yoshida R. Ichimura K. Shinozaki K. (2011). Calmodulin-dependent activation of MAP kinase for ROS homeostasis in *Arabidopsis*. Mol. Cell 41, 649–660. 10.1016/j.molcel.2011.02.029, PMID: 21419340

[ref310] Tanaka K. Choi J. Cao Y. Stacey G. (2014). Extracellular ATP acts as a damage-associated molecular pattern (DAMP) signal in plants. Front. Plant Sci. 5:446. 10.3389/fpls.2014.00446, PMID: 25232361 PMC4153020

[ref311] Tanaka K. Gilroy S. Jones A. M. Stacey G. (2010). Extracellular ATP signaling in plants. Trends Cell Biol. 20, 601–608. 10.1016/j.tcb.2010.07.005, PMID: 20817461 PMC4864069

[ref312] Tang J. Han Z. Sun Y. Zhang H. Gong X. Chai J. (2015). Structural basis for recognition of an endogenous peptide by the plant receptor kinase PEPR1. Cell Res. 25, 110–120. 10.1038/cr.2014.161, PMID: 25475059 PMC4650589

[ref313] Tarr S. A. J. (1972). Principles of plant pathology. London: The MacMillan Press.

[ref314] Tavormina P. De Coninck B. Nikonorova N. De Smet I. Cammue B. P. A. (2015). The plant peptidome: an expanding repertoire of structural features and biological functions. Plant Cell 27, 2095–2118. 10.1105/tpc.15.00440, PMID: 26276833 PMC4568509

[ref315] Theodoulou F. L. Job K. Slocombe S. P. Footitt S. Holdsworth M. Baker A.. (2005). Jasmonic acid levels are reduced in COMATOSE ATP-binding cassette transporter mutants. Implications for transport of jasmonate precursors into peroxisomes. Plant Physiol. 137, 835–840. 10.1104/pp.105.059352, PMID: 15761209 PMC1065384

[ref316] Thines B. Katsir L. Melotto M. Niu Y. Mandaokar A. Liu G.. (2007). JAZ repressor proteins are targets of the SCF^COI1^ complex during jasmonate signalling. Nature 448, 661–665. 10.1038/nature05960, PMID: 17637677

[ref317] Thomas J. O. Travers A. A. (2001). HMG1 and 2, and related ‘architectural’ DNA-binding proteins. Trends Biochem. Sci. 26, 167–174. 10.1016/S0968-0004(01)01801-1, PMID: 11246022

[ref318] Toyota M. Spencer D. Sawai-Toyota S. Jiaqi W. Zhang T. Koo A. J.. (2018). Glutamate triggers long-distance, calcium-based plant defense signaling. Science 361, 1112–1115. 10.1126/science.aat7744, PMID: 30213912

[ref319] Tran D. Dauphin A. Meimoun P. Kadono T. Nguyen H. T. H. Arbelet-Bonnin D.. (2018). Methanol induces cytosolic calcium variations, membrane depolarization and ethylene production in *Arabidopsis* and tobacco. Ann. Bot. 122, 849–860. 10.1093/aob/mcy038, PMID: 29579139 PMC6215043

[ref320] Tripathi D. Tanaka K. (2018). A crosstalk between extracellular ATP and jasmonate signaling pathways for plant defense. Plant Signal. Behav. 13:e1432229. 10.1080/15592324.2018.1432229, PMID: 29370573 PMC6103277

[ref321] Tripathi D. Zhang T. Koo A. J. Stacey G. Tanaka K. (2018). Extracellular ATP acts on jasmonate signaling to reinforce plant defense. Plant Physiol. 176, 511–523. 10.1104/pp.17.01477, PMID: 29180381 PMC6108377

[ref322] Usami S. Banno H. Ito Y. Nishihama R. Machida Y. (1995). Cutting activates a 46-kilodalton protein kinase in plants. Proc. Natl. Acad. Sci. U. S. A. 92, 8660–8664. 10.1073/pnas.92.19.8660, PMID: 11607579 PMC41026

[ref323] Vadassery J. Reichelt M. Hause B. Gershenzon J. Boland W. Mithöfer A. (2012). CML42-mediated calcium signaling coordinates responses to *Spodoptera* herbivory and abiotic stresses in *Arabidopsis*. Plant Physiol. 159, 1150–1175. 10.1104/pp.112.198150, PMID: 22570470 PMC3387702

[ref324] Vega-Muñoz I. Feregrino-Pérez A. A. Torres-Pacheco I. Guevara-González R. G. (2018). Exogenous fragmented DNA acts as a damage-associated molecular pattern (DAMP) inducing changes in CpG DNA methylation and defence-related responses in *Lactuca sativa*. Funct. Plant Biol. 45, 1065–1072. 10.1071/FP18011, PMID: 32291005

[ref325] Vénéreau E. Ceriotti C. Bianchi M. E. (2015). DAMPs from cell death to new life. Front. Immunol. 6:422. 10.3389/fimmu.2015.00422, PMID: 26347745 PMC4539554

[ref326] Vincent T. R. Avramova M. Canham J. Higgins P. Bilkey N. Mugford S. T.. (2017). Interplay of plasma membrane and vacuolar ion channels, together with BAK1, elicits rapid cytosolic calcium elevations in *Arabidopsis* during aphid feeding. Plant Cell 29, 1460–1479. 10.1105/tpc.17.00136, PMID: 28559475 PMC5502460

[ref327] Vincill E. D. Bieck A. M. Spalding E. P. (2012). Ca^2+^ conduction by an amino acid-gated ion channel related to glutamate receptors. Plant Physiol. 159, 40–46. 10.1104/pp.112.197509, PMID: 22447719 PMC3375973

[ref328] Vodeneev V. Akinchits E. Sukhov V. (2015). Variation potential in higher plants: mechanisms of generation and propagation. Plant Signal. Behav. 10:e1057365. 10.1080/15592324.2015.1057365, PMID: 26313506 PMC4883923

[ref329] Vodeneev V. Orlova A. Morozova E. Orlova L. Akinchits E. Orlova O.. (2012). The mechanism of propagation of variation potentials in wheat leaves. J. Plant Physiol. 169, 949–954. 10.1016/j.jplph.2012.02.013, PMID: 22533926

[ref330] Wagner T. A. Kohorn B. D. (2001). Wall-associated kinases are expressed throughout plant development and are required for cell expansion. Plant Cell 13, 303–318. 10.1105/tpc.13.2.303, PMID: 11226187 PMC102244

[ref331] Wang L. Einig E. Almeida-Trapp M. Albert M. Fliegmann J. Mithöfer A.. (2018). The systemin receptor SYR1 enhances resistance of tomato against herbivorous insects. Nat. Plants 4, 152–156. 10.1038/s41477-018-0106-0, PMID: 29459726

[ref332] Wang C. Huang X. Li Q. Zhang Y. Li J.-L. Mou Z. (2019). Extracellular pyridine nucleotides trigger plant systemic immunity through a lectin receptor kinase/BAK1 complex. Nat. Commun. 10:4810. 10.1038/s41467-019-12781-7, PMID: 31641112 PMC6805918

[ref333] Wang Y. H. Irving H. R. (2011). Developing a model of plant hormone interactions. Plant Signal. Behav. 6, 494–500. 10.4161/psb.6.4.14558, PMID: 21406974 PMC3142376

[ref334] Wang Q.-W. Jia L.-Y. Shi D.-L. Wang R.-F. Lu L.-N. Xie J.-J.. (2019). Effects of extracellular ATP on local and systemic responses of bean (*Phaseolus vulgaris* L) leaves to wounding. Biosci. Biotechnol. Biochem. 83, 417–428. 10.1080/09168451.2018.1547623, PMID: 30458666

[ref335] Wang C. Zhou M. Zhang X. Yao J. Zhang Y. Mou Z. (2017). A lectin receptor kinase as a potential sensor for extracellular nicotinamide adenine dinucleotide in *Arabidopsis thaliana*. eLife 6:e25474. 10.7554/eLife.25474, PMID: 28722654 PMC5560858

[ref336] Wasternack C. Feussner I. (2018). The oxylipin pathways: biochemistry and function. Annu. Rev. Plant Biol. 69, 363–386. 10.1146/annurev-arplant-042817-040440, PMID: 29166128

[ref337] Wen F. White G. J. VanEtten H. D. Xiong Z. Hawes M. C. (2009). Extracellular DNA is required for root tip resistance to fungal infection. Plant Physiol. 151, 820–829. 10.1104/pp.109.142067, PMID: 19700564 PMC2754639

[ref338] Wild M. Davière J. -M. Cheminant S. Regnault T. Baumberger N. Heintz D.. (2012). The *Arabidopsis* DELLA *RGA-LIKE3* is a direct target of MYC2 and modulates jasmonate signaling responses. Plant Cell 24, 3307–3319. 10.1105/tpc.112.101428, PMID: 22892320 PMC3462633

[ref339] Will T. Tjallingii W. F. Thönnessen A. van Bel A. J. E. (2007). Molecular sabotage of plant defense by aphid saliva. Proc. Natl. Acad. Sci. U. S. A. 104, 10536–10541. 10.1073/pnas.0703535104, PMID: 17553961 PMC1965548

[ref340] Williams C. Fernández-Calvo P. Colinas M. Pauwels L. Goossens A. (2019). Jasmonate and auxin perception: how plants keep F-boxes in check. J. Exp. Bot. 70, 3401–3414. 10.1093/jxb/erz272, PMID: 31173086

[ref341] Wolf S. (2017). Plant cell wall signalling and receptor-like kinases. Biochem. J. 474, 471–492. 10.1042/BCJ20160238, PMID: 28159895

[ref342] Wolf S. Hématy K. Höfte H. (2012). Growth control and cell wall signaling in plants. Annu. Rev. Plant Biol. 63, 381–407. 10.1146/annurev-arplant-042811-105449, PMID: 22224451

[ref343] Wu F. Chi Y. Jiang Z. Xu Y. Xie L. Huang F.. (2020). Hydrogen peroxide sensor HPCA1 is an LRR receptor kinase in *Arabidopsis*. Nature 578, 577–581. 10.1038/s41586-020-2032-3, PMID: 32076270

[ref344] Wu J. Hettenhausen C. Meldau S. Baldwin I. T. (2007). Herbivory rapidly activates MAPK signaling in attacked and unattacked leaf regions but not between leaves of *Nicotiana attenuata*. Plant Cell 19, 1096–1122. 10.1105/tpc.106.049353, PMID: 17400894 PMC1867352

[ref345] Wu S. -W. Kumar R. Iswanto A. B. B. Kim J. Y. (2018). Callose balancing at plasmodesmata. J. Exp. Bot. 69, 5325–5339. 10.1093/jxb/ery317, PMID: 30165704

[ref346] Wu S. Peiffer M. Luthe D. S. Felton G. W. (2012). ATP hydrolyzing salivary enzymes of caterpillars suppress plant defenses. PLoS One 7:e41947. 10.1371/journal.pone.0041947, PMID: 22848670 PMC3405022

[ref347] Xu B. Cheval C. Laohavisit A. Hocking B. Chiasson D. Olsson T. S. G.. (2017). A calmodulin-like protein regulates plasmodesmal closure during bacterial immune responses. New Phytol. 215, 77–84. 10.1111/nph.14599, PMID: 28513846 PMC5488192

[ref348] Yamada K. Yamashita-Yamada M. Hirase T. Fujiwara T. Tsuda K. Hiruma K.. (2016). Danger peptide receptor signaling in plants ensures basal immunity upon pathogen-induced depletion of BAK1. EMBO J. 35, 46–61. 10.15252/embj.201591807, PMID: 26574534 PMC4718002

[ref349] Yamaguchi Y. Huffaker A. Bryan A. C. Tax F. E. Ryan C. A. (2010). PEPR2 is a second receptor for the Pep1 and Pep2 peptides and contributes to defense responses in *Arabidopsis*. Plant Cell 22, 508–522. 10.1105/tpc.109.068874, PMID: 20179141 PMC2845411

[ref350] Yamaguchi Y. Pearce G. Ryan C. A. (2006). The cell surface leucine-rich repeat receptor for *At*Pep1, an endogenous peptide elicitor in *Arabidopsis*, is functional in transgenic tobacco cells. Proc. Natl. Acad. Sci. U. S. A. 103, 10104–10109. 10.1073/pnas.0603729103, PMID: 16785433 PMC1502513

[ref351] Yan C. Fan M. Yang M. Zhao J. Zhang W. Su Y.. (2018). Injury activates Ca^2+^/calmodulin-dependent phosphorylation of JAV1-JAZ8-WRKY51 complex for jasmonate biosynthesis. Mol. Cell 70, 136–149. 10.1016/j.molcel.2018.03.013, PMID: 29625034

[ref352] Yan Y. Stolz S. Chételat A. Reymond P. Pagni M. Dubugnon L.. (2007). A downstream mediator in the growth repression limb of the jasmonate pathway. Plant Cell 19, 2470–2483. 10.1105/tpc.107.050708, PMID: 17675405 PMC2002611

[ref353] Yan S. Wang W. Marqués J. Mohan R. Saleh A. Durrant W. E.. (2013). Salicylic acid activates DNA damage responses to potentiate plant immunity. Mol. Cell 52, 602–610. 10.1016/j.molcel.2013.09.019, PMID: 24207055 PMC3863363

[ref354] Yang T. -H. Lenglet-Hilfiker A. Stolz S. Glauser G. Farmer E. E. (2020). Jasmonate precursor biosynthetic enzymes LOX3 and LOX4 control wound-response growth restriction. Plant Physiol. 184, 1172–1180. 10.1104/pp.20.00471, PMID: 32669418 PMC7536668

[ref355] Ye Z. -W. Lung S. -C. Hu T. -H. Chen Q. -F. Suen Y. -L. Wang M.. (2016). Arabidopsis acyl-CoA-binding protein ACBP6 localizes in the phloem and affects jasmonate composition. Plant Mol. Biol. 92, 717–730. 10.1007/s11103-016-0541-0, PMID: 27645136

[ref356] Yin H. Yan B. Sun J. Jia P. Zhang Z. Yan X.. (2012). Graft-union development: a delicate process that involves cell-cell communication between scion and stock for local auxin accumulation. J. Exp. Bot. 63, 4219–4232. 10.1093/jxb/ers109, PMID: 22511803 PMC3398452

[ref357] Yip Delormel T. Boudsocq M. (2019). Properties and functions of calcium-dependent protein kinases and their relatives in *Arabidopsis thaliana*. New Phytol. 224, 585–604. 10.1111/nph.16088, PMID: 31369160

[ref358] Yoshiyama K. O. Aoshima N. Takahashi N. Sakamoto T. Hiruma K. Saijo Y.. (2020). SUPPRESSOR OF GAMMA RESPONSE 1 acts as a regulator coordinating crosstalk between DNA damage response and immune response in *Arabidopsis thaliana*. Plant Mol. Biol. 103, 321–340. 10.1007/s11103-020-00994-0, PMID: 32277429

[ref359] Yoshiyama K. Conklin P. A. Huefner N. D. Britt A. B. (2009). Suppressor of gamma response 1 (*SOG1*) encodes a putative transcription factor governing multiple responses to DNA damage. Proc. Natl. Acad. Sci. U. S. A. 106, 12843–12848. 10.1073/pnas.0810304106, PMID: 19549833 PMC2722309

[ref360] Zander M. Lewsey M. G. Clark N. M. Yin L. Bartlett A. Saldierna Guzmán J. P.. (2020). Integrated multi-omics framework of the plant response to jasmonic acid. Nat. Plants 6, 290–302. 10.1038/s41477-020-0605-7, PMID: 32170290 PMC7094030

[ref361] Zhang L. Gleason C. (2020). Enhancing potato resistance against root-knot nematodes using a plant-defence elicitor delivered by bacteria. Nat. Plants 6, 625–629. 10.1038/s41477-020-0689-0, PMID: 32514146

[ref362] Zhang X. Mou Z. (2009). Extracellular pyridine nucleotides induce PR gene expression and disease resistance in *Arabidopsis*. Plant J. 57, 302–312. 10.1111/j.1365-313X.2008.03687.x, PMID: 18798871

[ref363] Zhang G. Zhao F. Chen L. Pan Y. Sun L. Bao N.. (2019). Jasmonate-mediated wound signalling promotes plant regeneration. Nat. Plants 5, 491–497. 10.1038/s41477-019-0408-x, PMID: 31011153

[ref364] Zhang Y. Zheng L. Hong J. H. Gong X. Zhou C. Pérez-Pérez J. M.. (2016). TOPOISOMERASE1α acts through two distinct mechanisms to regulate stele and columella stem cell maintenance. Plant Physiol. 171, 483–493. 10.1104/pp.15.01754, PMID: 26969721 PMC4854680

[ref365] Zheng X. Kang S. Jing Y. Ren Z. Li L. Zhou J. -M.. (2018). Danger-associated peptides close stomata by OST1-independent activation of anion channels in guard cells. Plant Cell 30, 1132–1146. 10.1105/tpc.17.00701, PMID: 29716993 PMC6002199

[ref366] Zhou F. Emonet A. Dénervaud Tendon V. Marhavy P. Wu D. Lahaye T.. (2020). Co-incidence of damage and microbial patterns controls localized immune responses in roots. Cell 180, 440–453. 10.1016/j.cell.2020.01.013, PMID: 32032516 PMC7042715

[ref367] Zhou W. Lozano-Torres J. L. Blilou I. Zhang X. Zhai Q. Smant G.. (2019). A jasmonate signaling network activates root stem cells and promotes regeneration. Cell 177, 942–956. 10.1016/j.cell.2019.03.006, PMID: 30955889

[ref368] Zhu P. Yu X. -H. Wang C. Zhang Q. Liu W. McSweeney S.. (2020). Structural basis for Ca^2+^-dependent activation of a plant metacaspase. Nat. Commun. 11:2249. 10.1038/s41467-020-15830-8, PMID: 32382010 PMC7206013

[ref369] Zimmermann M. R. Maischak H. Mithöfer A. Boland W. Felle H. H. (2009). System potentials, a novel electrical long-distance apoplastic signal in plants, induced by wounding. Plant Physiol. 149, 1593–1600. 10.1104/pp.108.133884, PMID: 19129416 PMC2649404

[ref370] Zimmermann M. R. Mithöfer A. Will T. Felle H. H. Furch A. C. U. (2016). Herbivore-triggered electrophysiological reactions: candidates for systemic signals in higher plants and the challenge of their identification. Plant Physiol. 170, 2407–2419. 10.1104/pp.15.01736, PMID: 26872949 PMC4825135

